# Arterial vasodilation drives convective fluid flow in the brain: a poroelastic model

**DOI:** 10.1186/s12987-022-00326-y

**Published:** 2022-05-15

**Authors:** Ravi Teja Kedarasetti, Patrick J. Drew, Francesco Costanzo

**Affiliations:** 1 Department of Engineering Science and Mechanics, Pennsylvania State University, University Park, PA USA; 2 Center for Neural Engineering, Pennsylvania State University, University Park, PA USA; 3 Department of Biomedical Engineering, Pennsylvania State University, University Park, PA USA; 4 Department of Neurosurgery, Pennsylvania State University, University Park, PA USA; 5 Department of Mathematics, Pennsylvania State University, University Park, PA USA

## Abstract

The movement of fluid into, through, and out of the brain plays an important role in clearing metabolic waste. However, there is controversy regarding the mechanisms driving fluid movement in the fluid-filled paravascular spaces (PVS), and whether the movement of metabolic waste in the brain extracellular space (ECS) is primarily driven by diffusion or convection. The dilation of penetrating arterioles in the brain in response to increases in neural activity (neurovascular coupling) is an attractive candidate for driving fluid circulation, as it drives deformation of the brain tissue and of the PVS around arteries, resulting in fluid movement. We simulated the effects of vasodilation on fluid movement into and out of the brain ECS using a novel poroelastic model of brain tissue. We found that arteriolar dilations could drive convective flow through the ECS radially outward from the arteriole, and that this flow is sensitive to the dynamics of the dilation. Simulations of sleep-like conditions, with larger vasodilations and increased extracellular volume in the brain showed enhanced movement of fluid from the PVS into the ECS. Our simulations suggest that both sensory-evoked and sleep-related arteriolar dilations can drive convective flow of cerebrospinal fluid not just in the PVS, but also into the ECS through the PVS around arterioles.

**Supplementary Information:**

The online version contains supplementary material available at 10.1186/s12987-022-00326-y.

## Introduction

The circulation of cerebrospinal fluid (CSF) is thought to play the important role of clearing harmful solutes like amyloid-β from the brain [14, 21, 69, 118, 119]. The accumulation of these solutes in the brain extracellular spaces (ECS) has been linked to neurodegenerative diseases like Alzheimer’s [44, 93] and cerebral amyloid angiopathy [123, 124]. The fluid-filled paravascular spaces (PVS) surrounding arteries and arterioles in the brain could provide a low resistance pathway for fluid and solute exchange between the CSF in the subarachnoid space (SAS) and the interstitial fluid (ISF) in the ECS, thereby playing a key role in the clearance of harmful metabolites. Studies in mice have shown that dyes injected into the cisterna magna or in the ventricles of the brain enter the ECS of the cerebral cortex primarily along the PVS of arterioles, suggesting that the PVS is the preferred pathway of solute exchange between CSF and ISF [54–56, 82]. However, the nature and drivers of solute transport through the PVS remains controversial [1, 4, 50, 57, 60–62, 78, 88, 101, 102]. While experimental data is key to understanding the fluid flow in the brain as it is direct physical evidence, there are certain inherent limitations and artifacts to experimental approaches [79]. For example, with the currently available fluid tracing methods, fluid motion in the PVS and ECS has only been observed under Ketamine/Xylazine anesthesia but not in the awake state [78, 86], and the insertion of glass pipettes and needles etc. into the brain parenchyma can appreciably alter the fluid flow in the brain [77]. In view of these limitations, mathematical modeling can be a valuable auxiliary tool in understanding the mechanisms driving fluid flow and solute transport through the PVS.

**Fig. 1:**
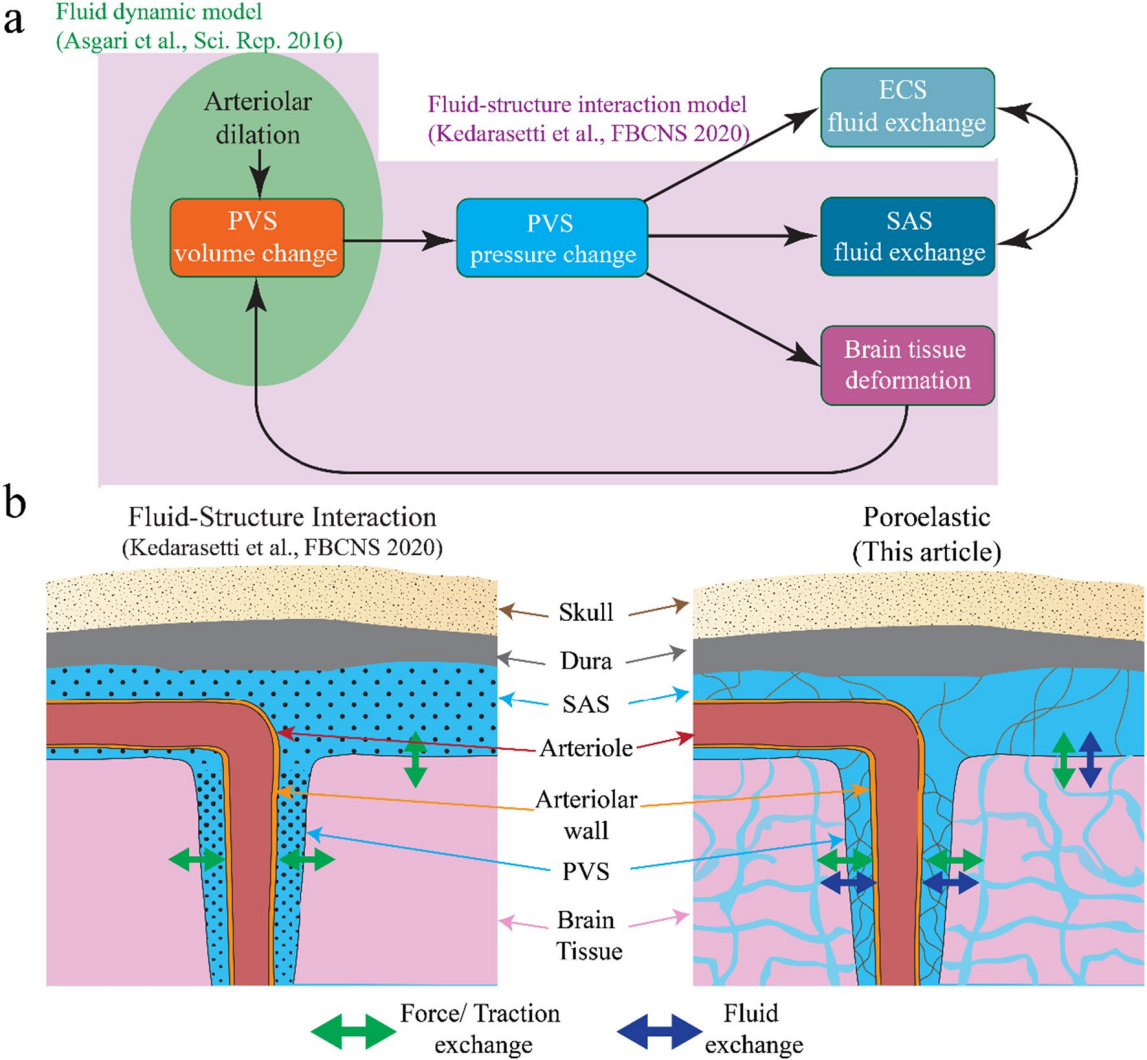
Schematic showing the working of a poroelastic model of the PVS, SAS and the brain tissue. **a.** Flowchart showing the full range of physics at play between the PVS, SAS, brain tissue, and the ECS that can be simulated by a poroelastic model. The field in green represents the physics that traditional fluid dynamic models capture (cf. [4]. The field light purple (which contains the field in green) represents the physics captured by traditional fluid–structure interaction models (cf. [61]). The model presented in this paper extends the physics captured within the light purple field to also include the physics represented by the arrows outside said field. **b.** The advantages of using a poroelastic model over a traditional fluid–structure interaction model. In our previous fluid–structure interaction model we only simulated the fluid phase in the PVS and the SAS (shown by black dots). By contrast, with a poroelastic model we can also simulate the elasticity of the connective tissue and, more importantly, the fluid flow and transport through the ECS. These differences mean that a poroelastic model can simulate fluid exchange between the brain parenchyma and other fluid spaces along with the force exchange that can be simulated by a fluid–structure interaction model

Several mathematical models have attempted to understand the nature and drivers of fluid and solute transport through the PVS [3, 4, 10, 23, 50, 61, 62, 73, 92, 106, 116]. However, the majority of published models of transport through the PVS have only simulated the fluid dynamics in the PVS in isolation [4, 10, 116]. These models only simulate fluid flow due to volume changes in the PVS that are directly driven by the movement the arteriolar walls (green region in Fig. 1a). However, the effect of pressure changes in the PVS on the deformation of the surrounding ultrasoft brain tissue [16] is almost never taken into account, thereby ignoring the feedback effect that volume and shape changes of the PVS has on the very geometry within which fluid flow occurs. In recent work [61], we addressed this limitation by using fluid–structure interaction models to simulate the effect of brain elasticity on fluid exchange between the PVS and the SAS (pink region in Fig. 1a). The fluid–structure interaction models we used assumed that only one phase (fluid or solid) was present in the spatial domain said phase occupied. That is, the elastic response of the connective tissue in the fluid-filled spaces (SAS and PVS) and the fluid flow in the ECS were not simulated.

In this study, we improve on our previous modeling of transport through the PVS and brain by using 3D poroelastic models. Poroelastic models based on mixture theory [12, 20, 13] can simultaneously simulate the solid and fluid phases in the same spatial domain, and the interactions between them. Using poroelastic models, simulations can be made of the volume changes of the PVS due to arteriolar wall movements and the resulting pressure changes in the PVS, which can drive fluid exchange between the PVS and the SAS or the ECS, and deform the brain tissue. Moreover, the poroelastic models can capture fluid exchange between the ECS and the SAS (Fig. 1a). Another way of thinking about the advantage of using a poroelastic model over a traditional fluid–structure interaction model is that while fluid–structure interaction models can only simulate the force transfer between the fluid filled regions (the SAS and the PVS) and the brain parenchyma, poroelastic models can additionally simulate the fluid-mass transfer between the fluid-filled regions and the brain parenchyma (Fig. 1b). To the best of our knowledge, the only published model simulating flow through the PVS and the ECS simultaneously while accounting for the deformation of the brain tissue is by Romanò et al*.*, [90]. These authors considered an axisymmetric model of the PVS in which flow is studied with a hierarchical expansion of the equations from lubrication theory. While deformability of the brain tissue was accounted by way of a linear elastic model, flow in the brain was not modeled using a nonlinear poroelasticity as we have done in this study. Another, and perhaps more important, difference between our study and that by Romanò et al*.* [90], is the scale of the physical domain that is considered. Romanò et al*.* [90] consider a range of values for the reference PVS length the possibilty of flow along the entirety of the vascular tree, we have chosen to limit our analysis to the characteristic length of arterioles before they branch into capillaries. Our choice is consistent with that of our previous studies [61, 62]. Our choice is based, first and foremost, on our experience in the lab in that even under isoflurane anesthesia, no measurable pulsation of penetrating arterioles was observed [61]. This consideration is in addition to the fact that there is considerable uncertainty on the actual anatomy of the PVS, which is not a settled element of the glymphatic system hypothesis. This said, the fundamental novelty of this study is that we consider an alternative mechanism to peristalsis as the driver of flow through the PVS and into the ECS, as we discuss next.

Using 3D poroelastic models, we considered two modes of solute transport through the PVS: dispersion and convection. Dispersion could improve solute transport over diffusion by oscillatory fluid exchange between the PVS and the SAS or the PVS and the ECS, while convection can drive directional fluid and solute transport from the SAS to the ECS, via the PVS. Several published models of transport through the PVS suggest that dispersion is the main mechanism of solute transport through the PVS [4, 61, 73]. Dispersion-based solute transport is theoretically possible by any oscillatory movement of the arteriolar walls, like heartbeat-driven pulsations, intrinsic vasomotion of arteries [22, 120] and vasodilation due to increased neural activity, which have all been proposed as possible drivers of CSF flow [7, 49, 78, 112]. However, while some fluid dynamic calculations suggest the possibility of appreciable enhancement of solute transport through dispersion [63], others suggest that dispersion with purely oscillatory flow would be a very ineffective means of solute transport in the PVS and convection (even with low mean fluid velocities, of the order of $0.1\, \upmu {\text{m}}/{\text{s}}$) would result in faster solute transport [109]. Therefore, in this study, we focus on solving the simpler problem of convective fluid flow from the PVS to the ECS and the possible drivers of this convective flow, and leave the implications on dispersive transport to future studies.

In this study, we demonstrate the possibility of convective transport through the PVS driven by a combination of the non-linear flow response of the fluid spaces in the brain and asymmetry in the waveform of arterial wall motions. The possibility of directional fluid flow through the PVS has been previously explored through mathematical models and numerical simulations [4, 10, 23, 62, 92, 116]. However, most of the published models only considered the peristaltic motion of arteries driven by heartbeat pulsations as the possible driver of convectional transport. While it is theoretically possible to drive directional CSF flow by peristaltic pumping [10, 116], models using realistic dimensions and boundary conditions representing the anatomy of the PVS [4, 23, 62] suggest that heartbeat-driven pulsations of arteries drive mostly oscillatory flow in the mouse brain with negligible directional fluid flow. To the best of our knowledge, this study is the first to consider an alternative mechanism to peristalsis for driving convective transport through the PVS. Although the driving force in peristaltic pumping and the mechanism presented in this study is the movement of walls of the channel, the mechanism that leads to directional fluid movement is very different. In peristaltic pumping, a net directional fluid movement is achieved through the directionality in the flow resistance of a channel, which is only possible by a travelling wave of wall movements. In the mechanism proposed here, the directional fluid movement is achieved by the non-linear transient and asymmetric flow resistance of the porous spaces that surround arteries, which does not require a traveling wave of arterial wall motion. Previous studies have considered alternative mechanisms like differential reflection of the peristaltic wave in different layers of the arteriolar wall [19], directional resistors [95] and directional permeability [25] for the directional fluid movement in the fluid-filled basement membrane of arteries (perivascular spaces) but not for the PVS between the arterial wall and the astrocytic endfeet.

The role of arterial dilations and subsequent return to baseline during functional hyperemia in driving fluid and solute transport through the PVS will be the major focus of this study. Functional hyperemia [53] is the dilation of arteries and arterioles in the brain in regions of increased neural activity, potentially driven by a subset of neurons. Though it is often stated that functional hyperemia is required to match the brain’s energetic demands [67], this is not the case, and the underlying physiological purpose of functional hyperemia remains unclear. The hypothesis that functional hyperemia drives PVS solute transport has received some attention recently, with support from both experiments [49, 112] and theoretical models [61]. Using our 3D poroelastic models, we tried to understand how different characteristics of functional hyperemia affect transport through the PVS. Our models showed that the temporal characteristics of functional hyperemia, which usually consist of a rapid dilation of arterioles (reaching the peak dilation within two seconds) followed by a slower return to resting diameter over several seconds [29, 41, 45, 100] could drive convective fluid flow. The models also showed that hyperemia during sleep, which has arteriolar dilations several times larger than those during the awake state [9, 111], combined with the increased extracellular volume in the brain [122] could explain the larger solute transport in the brain parenchyma observed during sleep [42, 122]. The models also suggest that the low frequency oscillations in vessel dilation during neural activity and sleep play a major role in transport through the PVS.

### Model assumptions

**Fig. 2:**
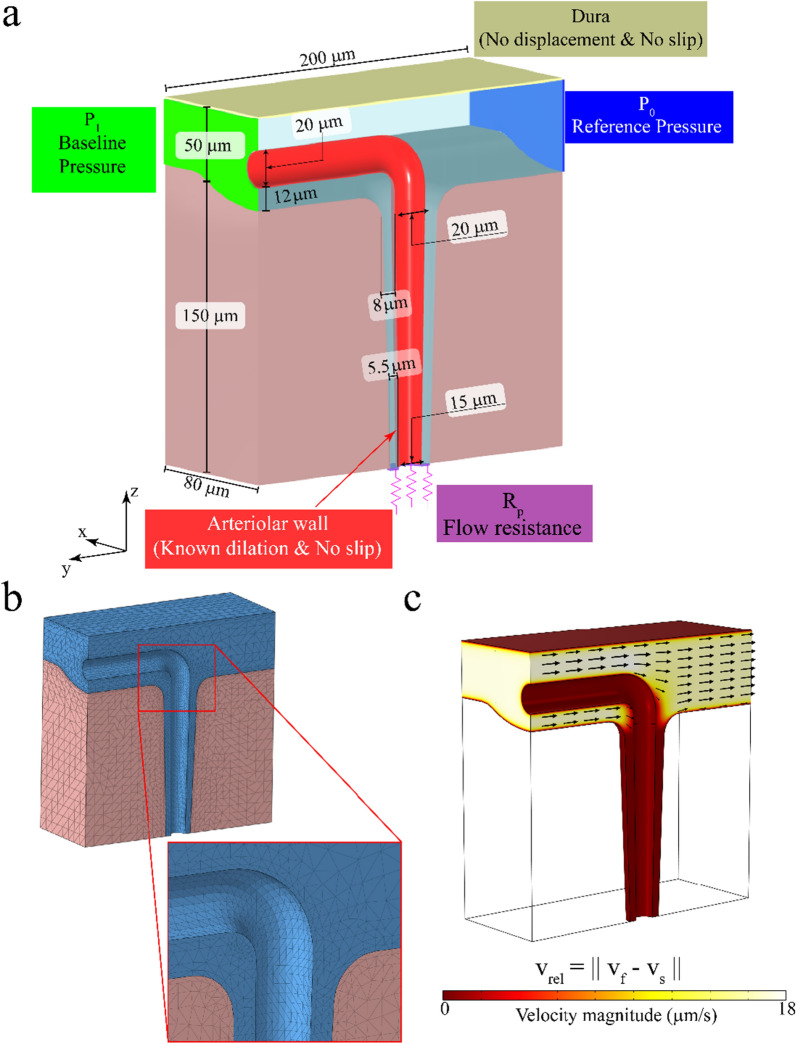
Geometry, boundary conditions and discretization of the model. **a.** The geometry of the model showing the two domains, with the dimensions and boundary conditions. Solid displacement and fluid velocity were prescribed at the red- and cream-colored surfaces. Pressure-like tractions were prescribed on the green- and blue-colored surfaces. Flow resistance (Robin) boundary conditions were prescribed on the purple-colored surface. Symmetry boundary conditions were prescribed on all other surfaces. **b.** Tetrahedral mesh used for the finite element model. A fine mesh, with elements of $2 \,\upmu \mathrm{m}$ were used near the regions where no-slip boundary conditions were prescribed and at the interface between the two domains. The mesh size was gradually increased to $10\,\upmu\mathrm{m}$**. c** The fluid flow in the SAS at the baseline state, which is a result of the pressure difference applied across the ends (green- and blue-colored surfaces in **a**)

The geometry of the model was created to represent the anatomy of a single penetrating arteriole in the mouse cortex, while keeping the shape relatively simple. The dimensions of the model geometry are shown in Fig. 2a. The entire geometry had a size of ($x\times y\times z$) $80\, \upmu {\text{m}}\times 200\, \upmu {\text{m}}\times 200\, \upmu {\text{m}}$, with the $z$ direction being perpendicular to the pial surface. The model was composed of two domains, one representing the fluid-filled SAS and the PVS (translucent blue in Fig. 2a) and the other representing the poroelastic brain tissue (pink in Fig. 2a). To keep the geometry simple, the model simulated a segment of the brain from the cortical surface to $150\, \upmu {\text{m}}$ in depth ($z$ direction), below which arterioles usually branch out into smaller arterioles or capillaries [11, 38, 51]. The dimensions of the geometry in the $x$ and $y$ directions were chosen to represent half of the typical separation between arterioles in the cortex [2, 38, 84, 97]. The SAS of the model had a nominal width of $50\, \upmu {\text{m}}$ [17, 18]. The part of the geometry representing the SAS and the PVS was built with a cavity representing an arteriole penetrating into the brain. The segment of the arteriole passing through the SAS had a diameter of $20\, \upmu {\text{m}}$ [26, 96], with its long axis along the $y$-axis of the model. The arteriole was assumed to penetrate into the brain tissue along the $z$-axis, with its diameter tapering down to $15\, \upmu {\text{m}}$ at $150\, \upmu {\text{m}}$ below the brain surface of the brain. The PVS surrounding the arteriole was assumed to be an annular region with a width of $8\, \upmu \mathrm{m}$ near the surface of the brain and $5.5\, \upmu {\text{m}}$ at $150\, \upmu {\text{m}}$ below the brain surface. The dimensions of the PVS were taken from experimentally-determined values from published imaging data [54, 78, 91]. For the geometry of the PVS, a relatively simple annular shape was chosen instead of a more realistic eccentric and elliptical annular shape [81] to avoid further complicating the model by increasing the number of unknown parameters (like eccentricity), or by adding a cumbersome boundary condition at the common interface of the arteriole, the PVS, and the brain tissue. All the sharp corners in the model geometry were smoothened by using a circular fillet. The geometry was sliced in half at the $yz$ plane ($x=0$) to reduce the size of the calculations using symmetry boundary conditions (see the section on boundary conditions in Methods). The model was oriented so that the origin $\left(\mathrm{0,0},0\right)$ was on the axis of the vessel and at the bottom surface of the brain parenchyma. A tetrahedral mesh was created for the half section with elements of thickness $2\, \upmu {\text{m}}$ at the surfaces representing the arteriolar wall, the skull and the interface between the fluid-filled spaces and the brain tissue. The mesh size was gradually increased to $10\, \upmu {\text{m}}$ (Fig. 2b).

The constitutive models and the model parameters were chosen to capture the experimentally determined mechanics of the brain tissue and the surrounding fluid spaces. We use the superscript ^1^ to represent the parameters in the fluid-filled spaces, and ^2^ to represent the parameters in the brain tissue. An incompressible Darcy-Brinkman model was used for fluid flow through porous spaces, with a mass density (${\rho }_{f}^{*}$) of $1000 \,{\text{kg}}/{\text{m}}^{3}$ and viscosity (${\mu }_{f}$) of $0.001\,{\text{Pa}} \, {\text{s}}$ [104, 125]. The fluid permeability (we use the term permeability to designate the Darcy permeability, cf., e.g., [116]), ${k}_{s}^{2}$, of the brain tissue was assumed to be $2\times {10}^{-15} {\text{m}}^{2}$ (corresponding to values of hydraulic conductivity ${k}_{s}^{2}/{\mu }_{f}$ of $2\times {10}^{-12} {\text{m}}^{2}/\left({\text{Pa}} \, {\text{s}}\right)$ or $2\times {10}^{-9} {\text{c}}{\text{m}}^{4}/\left({\text{dyn}} \, {\text{s}}\right)$) based on experimental measurements [83, 103]. The permeability of the PVS, ${k}_{s}^{1}$ for $z <130\, \upmu\mathrm{m}$, was chosen to be $2\times {10}^{-14} {\text{m}}^{2}$, 10 times higher than that of the ECS. The SAS in the model is meant to represent a combination of the open (completely fluid-filled and not porous) PVS of surface arterioles [78, 81] and the porous SAS, and therefore, a higher permeability, ${k}_{s}^{1}$ for $z>150\,\upmu\mathrm{m}$, of $2\times {10}^{-12} {\text{m}}^{2}$ was used for the SAS. The permeability in the fluid-filled domain for $130\, \upmu {\text{m}}\le z\le 150\, \upmu {\text{m}}$ was transitioned using a function with continuous first and second derivatives ($step$ function in COMSOL Multiphysics). The brain tissue fluid volume fraction ${\zeta }_{{R}_{f}}^{2}$ was set to $0.2$ to represent the 20% of the brain volume occupied by the extracellular fluid, which is in the range of values measured with 2D and 3D electron microscopy without chemical fixation [50, 65], as well as measurements with real-time iontophoresis [122]. For the fluid-filled spaces, a higher fluid volume fraction ${\zeta }_{{R}_{f}}^{1}$ of $0.8$ was used. An incompressible neo-Hookean model was used for the solid phase. A shear modulus ${\mu }_{s}^{2}$ of $2\,{\text{kPa}}$ [15, 80, 117] was used for the brain tissue and a small shear modulus ${\mu }_{s}^{1}$ of $20\,{\text{Pa}}$ was used in the fluid-filled domain to represent the connective tissue in the fluid-filled spaces. A mass density of $1000\,\mathrm{kg}/\mathrm{m}^{3}$ was used for the solid phase [5]. In almost all simulations, a 20% dilation of arteriolar diameter was imposed with the temporal dynamics of the vasodilatory response to a brief ($1$–$2\,\mathrm{s}$ long) increase in neural activity [32, 61, 111].

The boundary conditions for the model were chosen to represent a segment of the cerebral cortex in an active region of the brain, i.e., a section of the cortex containing a dilating arteriole surrounded by other arterioles dilating with similar dynamics. This choice of boundary conditions (depicted in Fig. 2a) is apt for simulating both sleep and awake states, where arteries within a few square millimeter patch will dilate [111, 126, 127] simultaneously. The arteriolar wall motion was simulated by the radially outward solid displacement at the surface of the cavity representing the penetrating arteriole (red surface in Fig. 2a), and no-slip boundary conditions were used for the fluid. A small pressure difference was applied across the ends of the SAS in the form of traction forces on the fluid phases (${P}_{0}=0 \,{\text{mmHg}}$ on the blue surface and ${P}_{1}=0.01 \,{\text{mmHg}}$ on the green surface in Fig. 2a), to simulate the flow driven by the secretion of CSF (and possibly by arterial pulsations). The value of ${P}_{1}$ was chosen to achieve a maximum flow velocity of $20\, \upmu {\text{m}}/{\text{s}}$ around arterioles on the surface of the brain [7, 78], when the arteriole is at the baseline diameter (Fig. 2c). The bottom surface of the PVS ($z=0$) was assumed to be connected to the brain parenchyma and the PVS of smaller arterioles and therefore a flow resistance with a value of 10 times the flow resistance of the PVS was used (purple surface in Fig. 2a). This resistance was assumed to represent the flow resistance of all the pathways that fluid can flow through before re-entering the SAS, and therefore zero pressure was assumed beyond the resistor. Our assumed resistance of 10 times the PVS flow resistance is within the values used in the literature, namely between the values of zero flow resistance [4] and a capillary wall resistance of nearly 100 times the flow resistance of the PVS [113]. A zero-displacement condition for the solid phase and a no-slip boundary condition for the fluid phase were implemented on the top surface of the SAS ($z=200\, \upmu {\text{m}}$), which represents the skull-fixed dura. On all other free surfaces of the model, the solid displacement and fluid flow perpendicular to the surface were set to zero. For the $yz$-plane ($x=0$), this boundary condition reflects the symmetry assumption, while for the surfaces at $x=80\,\upmu\mathrm{m}$, $y=-100\,\upmu\mathrm{m}$ and $y=100\,\upmu\mathrm{m}$ the boundary condition reflects the assumption that the domains represented in the model are surrounded by similar structures experiencing similar arteriolar dilation. At the bottom surface of the brain tissue ($z=0$), the condition of no fluid flow perpendicular to the surface was deemed more apt than a flow resistance boundary condition, as the latter would set a spatially uniform, flow-dependent traction across the whole surface. At the interface between the two domains, mass and momentum continuity were maintained by special boundary conditions, usually referred to as jump conditions (see interface conditions in “Methods”).

## Results

### Functional hyperemia can drive directional fluid flow through the PVS

**Fig. 3:**
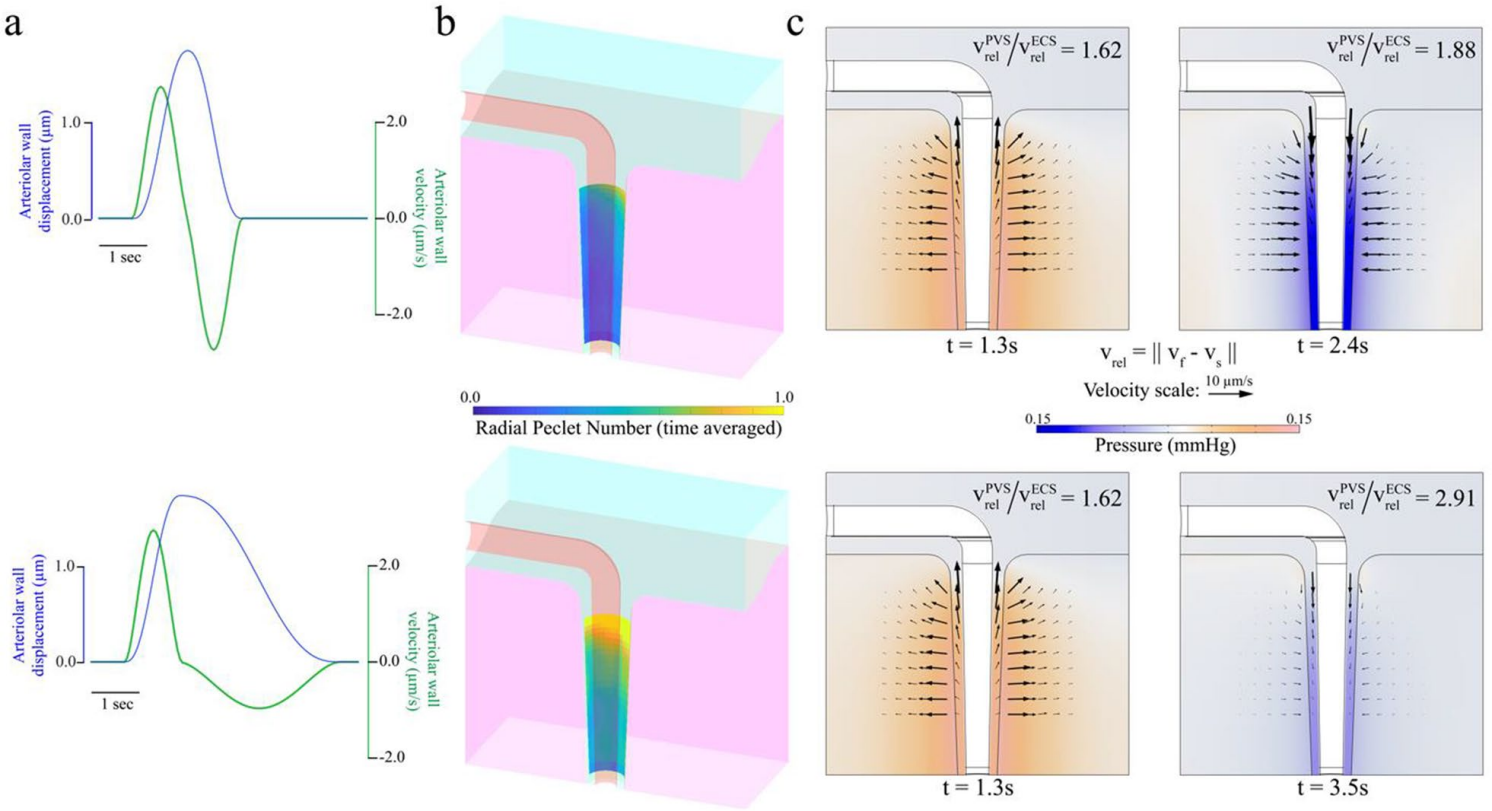
The asymmetric waveform of functional hyperemia can drive net directional fluid flow through the PVS. **a** The radially outward displacement (blue) and velocity (green) of the arteriolar wall for the case of symmetric dilation (top) and asymmetric dilation (bottom). **b** The time averaged radial Peclet numbers at the PVS-ECS interface as a result of symmetric (top) and (asymmetric) vasodilation. **c** The pressure and relative fluid velocity in the PVS and the ECS at the times of maximum radially outward and inward arteriolar wall velocity for symmetric (top) and asymmetric (bottom) dilation. The colors show the pressure value in mmHg and the arrows show the magnitude and direction of relative fluid flow. By comparing the ratio of the maximum relative velocity in the PVS and SAS, it can be seen with asymmetric vasodilation more fluid enters the ECS through the PVS than returns into the PVS through the ECS

We first examined the possibility of convective transport through the PVS driven by functional hyperemia and the factors contributing to this type of transport. Specifically, we quantified the contribution of the waveform of functional hyperemia to directional fluid flow through the PVS. To do this, we compared two modes of vasodilation, temporally symmetric and temporally asymmetric vasodilations. For symmetric vasodilation, the temporal waveform of arteriolar wall displacement resembles a Gaussian pulse, and the negative radial velocity of the arteriolar wall during the contraction of the vessel is equal and opposite to the positive radial velocity during dilation (Fig. 3a top). For the case of asymmetric dilation, the waveform of arteriolar wall displacement resembles that seen in functional hyperemia [27], with a sharper increase of vessel diameter at the beginning of the event, followed by a slow return to baseline (Fig. 3a bottom). In this case, the peak magnitude of the negative radial velocity of the arteriolar wall is roughly half the value of peak positive radial velocity. To quantify the convective flow driven by arteriolar dilation, we defined two time-averaged Peclet numbers (see "Methods", Peclet numbers), averaged over $10\,\mathrm{s}$ of simulation. The *axial* Peclet number, ${Pe}_{a}$, was defined based on the time-averaged relative fluid velocity (i.e., relative to the solid) through the bottom face of the PVS, and represents directional pumping by arteriolar wall motions in the traditional sense, similar to peristaltic pumping. The *radial* Peclet number, ${Pe}_{r}$, was defined based on the time-averaged radial component of the relative fluid velocity at the interface of the PVS and the brain tissue, and represents directional fluid flow into the ECS due to asymmetries in the flow resistances of the SAS-PVS-ECS system. It is important to acknowledge that the use of Peclet numbers in the current context is unconventional from a fluid dynamics perspective because the proposed Peclet numbers do not (in fact, cannot) arise from a nondimensionalization of our equations of motion. As such, these Peclet numbers are not flow indices (see “Methods”, Nondimensional numbers). Rather, they are meant to provide a way, if imperfect, to compare the convective flow we predict with a putative diffusion process, as we had already done in our previous work [61, 62]. For this putative process we select the diffusion coefficient of amyloid-β (${D}_{a\beta }$, see Table 1) and a characteristic length of $150\, \upmu {\text{m}}$ meant to represent the distance that a metabolite would have to traverse to go from an arterial to a venous PVS.

**Table 1:** Model parameters

Parameter	Value	Unit	Description	Sources
${\rho }_{f}^{*}$	$1000$	${\text{kg}}/{\text{m}}^{3}$	Fluid true density	[104, 125]
${\rho }_{s}^{*}$	$1000$	${\text{kg}}/{\text{m}}^{3}$	Solid true density	[5]
${\mu }_{f}$	$0.001$	${\text{Pa}} \, {\text{s}}$	Fluid dynamic viscosity	[104, 125]
${\zeta }_{{R}_{f}}^{2}$	$0.2$		Fluid volume fraction in tissue	[50, 65, 122]
${\zeta }_{{R}_{f}}^{1}$	$0.8$		Fluid volume fraction in PVS	
${k}_{s}^{2}$	$2\times {10}^{-15}$	${\text{m}}^{2}$	Fluid permeability of ECS	[83, 103]
${k}_{s}^{1}$	$2\times {10}^{-14}$	${\text{m}}^{2}$	Fluid permeability of PVS	
${\mu }_{s}^{2}$	$2$	${\text{kPa}}$	Shear Modulus of brain tissue	[15, 80, 117]
${\mu }_{s}^{1}$	$20$	${\text{Pa}}$	Shear modulus of connective tissue	
${R}_{0}$	$10$	$\upmu {\text{m}}$	Nominal vessel radius	[26, 96]
${D}_{a\beta }$	$1.4\times {10}^{-6}$	${\text{c}}{\text{m}}^{2}/{\text{s}}$	Diffusion coefficient of amyloid-β	[74, 110]
$\lambda$	$1.6$		Tortuosity of ECS	[105]

Arteriolar dilation with an asymmetric waveform resulted in appreciable radially outward fluid flow into the ECS through the PVS, while neither waveform resulted in directional fluid flow in the axial direction. The time-averaged radial Peclet numbers at the interface between the brain tissue and the PVS are shown in Fig. 3b. For the symmetric dilation waveform, the maximum time-averaged radial Peclet number was $1.11$ (Fig. 3b, top), while for the asymmetric waveform, the maximum time-averaged radial Peclet number was $2.07$ (Fig. 3b, top). The time-averaged axial Peclet number for the symmetric waveform was $0.0003$ and asymmetric waveform was $0.0012$. The axial Peclet number was calculated at the bottom surface ($z = 0$) to distinguish purely axial flow in the PVS from any fluid exchange between the ECS and the PVS. The low axial Peclet number does not preclude axial flow in the ECS or in the parts of the PVS closer to the surface. In fact, directional axial flow in the PVS close to the cortical surface is necessary for directional radial flow into the ECS. The low axial Peclet number might be a result of using a standing-wave dilation in our simulations rather than a traveling-wave dilation, although previously published mathematical models that used realistic dimensions of the PVS [4, 23, 62] suggest that traveling-wave dilations cannot drive directional fluid flow through the PVS alone. The directional fluid flow through the PVS and into to the ECS can also be inferred from the difference in the time-averaged axial flow velocity and Reynolds numbers in the PVS near the surface of the brain ($z=150\,\upmu {\rm m}$) and the bottom surface of the model ($z=0$). For the symmetric wave form, the time-averaged value of the axial flow velocity at the brain surface and bottom surface are − 2.17 μm/s and − 2.37E−04 μm/s respectively, with Reynolds numbers of − 1.73E-05 and − 1.90E−09. For the asymmetric wave form, the time-averaged value of the axial flow velocity at the brain surface and bottom surface are − 4.52 μm/s and − 6.11E−03 μm/s respectively, with Reynolds numbers of − 3.62E−05 and − 4.89E−08. The negative values of the fluid velocity and the Reynolds number indicate flow into the PVS, in the negative *z*-direction. The reason for more pronounced radially outward pumping from the asymmetric dilation compared to symmetric dilation is a result of the relative fluid velocity in the ECS during arteriolar dilation and contraction, and the ratio of the relative fluid velocities in the PVS to those in the ECS (Fig. 3c). In Fig. 3c, the arrows show the direction and magnitude of the relative fluid velocity in both the domains at the times of peak outward and inward velocity of the arteriolar wall. The ratio of the maximum velocity magnitudes (${v}_{rel}^{PVS}/{v}_{rel}^{ECS}$ in Fig. 3c) in the PVS and ECS indicate the ratio of fluid in the PVS being exchanged with the SAS and the ECS, respectively. For the case of symmetric dilation, this ratio of maximum velocity magnitudes is similar during dilation and contraction of the vessel, indicating that roughly the same amount of fluid leaving the PVS through the ECS returns into the PVS through the ECS. For the case of asymmetric dilation, the ratio of maximum velocity during contraction is nearly twice the ratio during dilation, indicating that during the slow contraction of the vessel, only a fraction of the fluid leaving the PVS through the ECS returns through the same path. Therefore, for each dilation and contraction there is a larger net directional flow from the SAS into the ECS through the PVS for the asymmetric dilation waveform compared to the symmetric dilation.

Note that the arrows plotted in Fig. 3c showing the relative fluid velocity are an indication of how far fluid travels in the PVS and the ECS, but not an indication of the amount of fluid entering or exiting the SAS. The amount of fluid moving can be better understood by examining the filtration velocities (Additional file 1: Fig. S2). Filtration velocity is the relative fluid velocity multiplied the fluid volume fraction (see Eq. in “Methods”), and is an indicator of the amount of fluid flow relative to the solid phase. The conservation of fluid-mass dictates that the fluid flowing through the interface between the ECS and the PVS needs to be conserved, which is why the component of filtration velocity perpendicular to the interface between the two domains remains continuous in Additional file 1: Fig. S2. The lower fluid volume fraction in the ECS means that for the same flowrate to be maintained, the fluid velocity in the ECS needs to be higher than the fluid velocity in the PVS. This is reflected in the higher magnitude relative fluid velocity in the ECS compared to the relative fluid velocity in the PVS, in Fig. 3c.

The fluid velocities in the PVS and the ECS seen in the model are a result of the difference in the response of poroelastic mixtures to volume and pressure changes. When an incompressible poroelastic mixture is subject to transient changes in the bounding volume of the solid skeleton, the fluid flow response is approximately linear with the volume changes, and the fluid flow rate closely follows the volume changes. However, when the mixture is subject to transient pressure changes, the fluid flow response is highly non-linear with respect to the pressure, and the fluid flow rate changes “lag” behind the pressure changes. To demonstrate this phenomenon, we created a simple 2D poroelastic model (Additional file 1: Fig. S1) of a square block of length $150\, \upmu {\text{m}}$. The top and bottom edges of the square are subject to zero solid displacement in the vertical direction and no-slip boundary conditions for the fluid. At the right end, the horizontal solid displacement is set to zero, while no traction is applied on the fluid phase. When a Gaussian pulse of displacement (along with no-slip boundary condition) is applied at the left end, the fluid flowrate at the right end follows the applied wall velocity (derivative of the displacement) waveform. In contrast, when a pressure-like traction is applied on the left end (on both solid and liquid phases), there is a clear lag between the fluid flowrate at the right end the pressure waveform. This kind of lag between applied pressure changes and the flow response of poroelastic solids has been observed in soils [94, 121]. When the arteriole dilates in our model, the fluid-filled domain is subject to volume changes, while the domain representing the parenchymal tissue is subject to pressure changes due to the volume changes in the fluid-filled domain. Therefore, while the PVS fluid outflow during arteriolar dilation occurs through both the SAS and the ECS, the PVS fluid inflow during the arteriolar contraction that follows dilation occurs more from the SAS, because the flow response through the ECS is lagging. This difference between the inflow and outflow pathways for PVS fluid is further enhanced with the asymmetric waveform, because the faster dilation drives larger pressure changes in the PVS, compared to the slower contraction that follows dilation.

The brain tissue deforms in the poroelastic model due to pressure changes in the PVS (Additional file 1: Fig. S3). This deformation of the brain tissue was also predicted by our fluid–structure interaction model and demonstrated by our in-vivo imaging data [61]. The radially outward displacement of the brain tissue relative to the displacement of the arteriolar wall in the 3D poroelastic model (Additional file 1: Fig. S3b, c) was smaller than the displacement predicted by the fluid–structure interaction model. There are two main reasons for this. First, the pressure in the PVS in the poroelastic model acts on both fluid and solid phases and drives fluid flow, unlike the fluid–structure interaction model where all the pressure is driving the deformation of the brain tissue. Second, the width of the PVS in this poroelastic model was in the higher range of possible values, while in our fluid–structure interaction model [61] the width of the PVS was in the lower range of possible values, and therefore, because of the larger cross-sectional area of the PVS, for the same volume of fluid displaced by the arteriolar dilation, the fluid velocities and the resulting pressure changes in this poroelastic model are smaller than those in the fluid–structure interaction model. In the poroelastic model, there was also a displacement of the brain tissue in the z-direction (towards the surface) during arteriolar dilation (Additional file 1: Fig. S3d, e). The displacement in the vertical direction was because of an “expansion” of the brain tissue when fluid from the PVS entered the ECS.

### Functional hyperemia can drive fluid penetration into the brain

A common method for experimentally visualizing fluid movement into the brain is to inject tracers (either a fluorescent dye or particles) into the “large” CSF chambers in the cranial space (cisterna magna or the ventricles) and observe their movement [49, 54, 70, 77]. To connect the results of the simulations to experimental observations we modeled the fluid movement driven by arteriolar dilation by adding fluid particle tracking to the poroelastic simulations. However, there is a key difference between the movement of the particles that we are simulating here and the movement of physical tracers. These simulated particles are merely passive tracers and do not have physical properties of their own. The simulated particles do not diffuse and have the same mobility as water, irrespective of the fluid volume and tortuosity changes in the PVS and the brain, unlike real tracers. The size of the particles in the figures do not correspond to the “real” size of the particles, they are for visualization purposes only. Diffusion equations were not added to the model to prevent further complicating the model. The physics at play is purely that of the computed fluid flow in the poroelastic mixture.

**Fig. 4:**
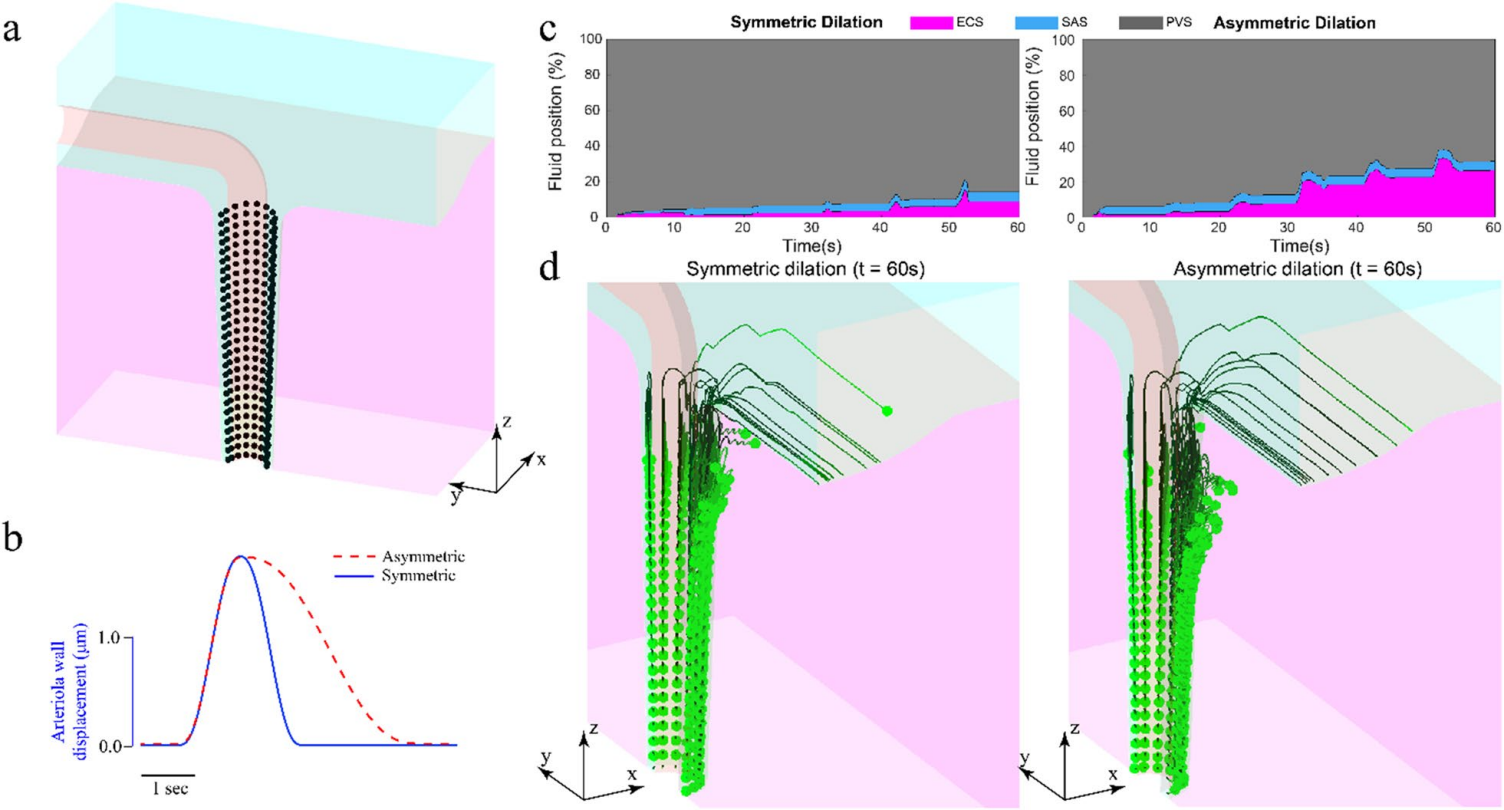
Functional hyperemia drives fluid penetration into the brain. **a** The initial position of particles used for particle tracking simulations. **b** The waveform of radial displacement of arteriolar displacement for symmetric and asymmetric dilation in the model. The asymmetric dilation waveform represents functional hyperemia. **c** The distribution of fluid position for 60 s of simulation with symmetric (left) and asymmetric (right) dilation waveform. Asymmetric dilation drives nearly three times (27%) PVS fluid movement into the brain compared to asymmetric dilation (9%). Both symmetric and asymmetric dilation drive similar PVS fluid movement into the SAS (5%). **d** The particle trajectories of fluid particles shown in (a). Asymmetric dilation moves PVS fluid deeper below the surface into the ECS and moves the fluid further into the brain

We started the particle tracking simulations with 243 equally spaced fluid particles (27 rings of 9 particles) in the PVS (Fig. 4a). The fluid particle motion was simulated for models with either symmetric or asymmetric vasodilation (Fig. 4b). These simulations had a duration of 60 s, with one vasodilation event occurred once every 10 s. At any given time in the simulation, the fluid particles were classified to be in the PVS, ECS or SAS based on their position. Area plots showing this distribution of particles (Fig. 4c), and 3D lines showing the particle trajectories for 60 s were plotted (Fig. 4d) to visualize and understand the physics of fluid motion through the fluid spaces surrounding a dilating arteriole.

The particle tracking simulations showed that the asymmetric waveform of functional hyperemia can drive appreciable fluid penetration into the ECS, with nearly three times (26.75%) the fluid particles moving from the PVS to the ECS compared to vasodilation with a symmetric waveform (9%) (Fig. 4c) over the same amount of time. The models suggest that for a symmetric waveform, only PVS fluid close to the surface of the brain penetrates into ECS, while for the asymmetric waveform the PVS fluid deeper in the brain penetrates into the ECS (Fig. 4d). Moreover, the radial distance to which the  fluid penetrates into the brain is larger for asymmetric dilation compared to symmetric dilation. The particle tracking simulations show that the PVS fluid that moves into the SAS is the same for symmetric and asymmetric dilation, which is expected, as the fluid velocity during dilation is same for both cases (Fig. 3c, left). Note that while the net flow of fluid is from the PVS into the ECS, there are times where the flow reverses.

The PVS fluid penetration into the ECS is not an artifact of the directional fluid flow imposed through the pressure difference across the two ends of the SAS (Fig. 2a). To verify this, we repeated the particle simulations with models where the pressure difference imposed across the ends of the SAS was 1/10th of the value used in the rest of the models, which resulted in a baseline flow peak relative fluid velocity of $2\, \upmu \text{m/s}$ in the SAS. Even in the case of reduced baseline fluid flow in the SAS, the time-averaged radial Peclet number (Additional file 1: Fig. S4a), PVS fluid position (Additional file 1: Fig. S4b) and PVS fluid trajectories (Additional file 1: Fig. S4c) were essentially unchanged, suggesting that the temporally asymmetric waveform of functional hyperemia can drive directional fluid flow from the PVS to the ECS. The maximum time-averaged radial Peclet number for the model with smaller pressure difference applied across the SAS was $1.93$. This result was expected because the pressure difference applied across the SAS drives very little flow through the PVS. Near the brain surface ($z=150\, \upmu {\text{m}}$), the values of time-averaged fluid velocity in the $z$-direction with $0.01 \,{\text{mmHg}}$ and $0.001 \,{\text{mmHg}}$ pressure differences across the SAS were $-4.523\, \upmu {\text{m}}/{\text{s}}$ and $-4.650\, \upmu {\text{m}}/{\text{s}}$, respectively, with a peak difference of $0.133\, \upmu {\text{m}}/{\text{s}}$ (Additional file 1: Fig. S4e).

### Sleep can enhance fluid penetration into the brain

An attractive hypothesis for the physiological purpose of sleep is to remove waste from the brain [58, 66, 122], as neurodegeneration is often preceded by sleep disruptions [71, 72]. Enhanced CSF movement has been observed during slow-wave (non-rapid eye movement) sleep in the brain of mice [122] and humans [35]. Large oscillations of cerebral blood volume (CBV) [9, 111] and increased extracellular volume [122], which also occur during sleep, have been proposed as the possible mechanisms for driving increased CSF movement during sleep. To determine the relative contributions of the changes in extracellular volume and larger arterial dilations during sleep to enhancing convection, we compared them individually and together to awake-like vasodilations. Using our particle tracking simulations, we examined the PVS fluid movement predicted by increased extracellular volume and large oscillations of CBV, to simulate the sleep state, and compared the resulting fluid movement in the PVS to that during a simulated awake state. The awake state was simulated with the default parameters in Table 1, and a 20% dilation of the artery with an asymmetric waveform, once every 10 s. For the simulated sleep state, the increased extracellular volume was simulated by increasing the fluid volume fraction in the domain representing the brain parenchyma from $0.2$ to $0.3$. The permeability of the ECS in the model was also increased from $2\times {10}^{-15} {\text{m}}^{2}$ to $4\times {10}^{-15} {\text{m}}^{2}$ to reflect the increased extracellular volume. The large CBV oscillations observed during sleep were simulated by 40% changes in vessel diameter [111], once every 10 s, with the same asymmetric waveform.

**Fig. 5:**
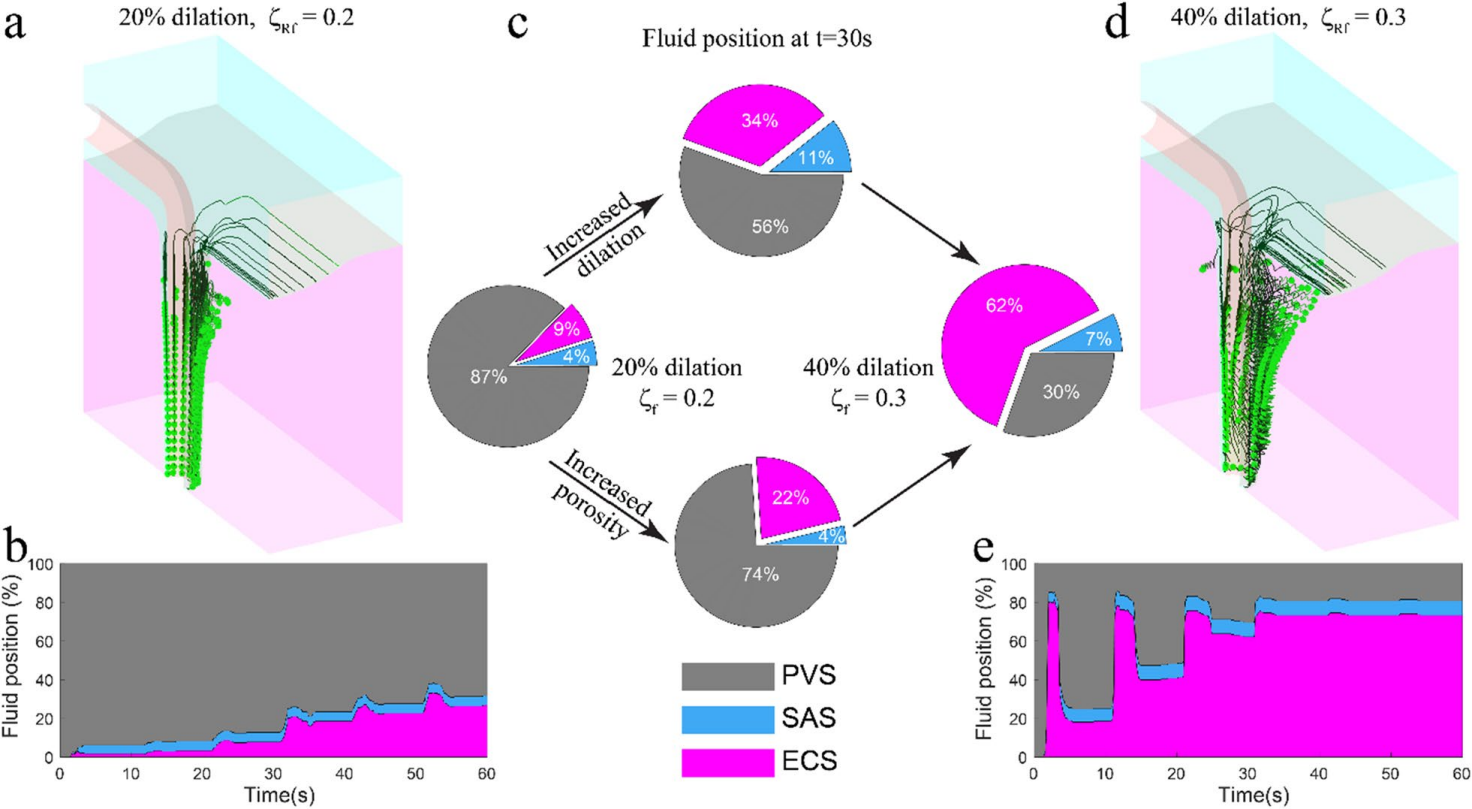
Sleep enhances fluid penetration into brain tissue. **a** The fluid particle trajectories for 60 s of simulation for the awake state (20% vessel dilation, once every 10 s with ${\zeta }_{{R}_{f}}^{2}=0.2$). **b** The fluid particle distribution for the awake state simulation. **c** The PVS fluid distribution at t = 30 s for simulated awake and sleep states, along with the cases where only the dilation and porosity are changed. The increase in porosity increased fluid movement into the ECS without affecting the fluid movement into the SAS, while larger dilations increased fluid movement to both SAS and ECS. **d** The fluid particle trajectories for 60 s of simulated sleep. The large amplitude vasodilation during sleep, combined with the increased porosity drives PVS fluid penetration into the brain. **e** The distribution of fluid position for 60 s of simulated sleep. **a**, **b** are repeated from the right side of Fig. 4d, c, respectively for better comparison with the sleep state

Our models suggest that both the increased extracellular volume (and permeability) and the larger CBV oscillations could contribute to larger PVS fluid movement into the ECS. Figure 5a shows the trajectories of fluid particles for the awake case at the end of 60 s and Fig. 5b shows the distribution of particle positions for 60 s. In contrast, the particle trajectories and positions during 60 s of simulated sleep (Fig. 5d, e, respectively) show that sleep enhances PVS fluid exchange with the SAS and the ECS. Sleep increases the amount of fluid entering the ECS from the PVS, and the distance of this fluid penetration into the ECS. To examine the contributions of changes in extracellular volume and amplitude of vasodilation to the PVS fluid movement independently, we plotted the fluid position at the end of 30 s of simulations, where only one of these changes were made to the awake case (Fig. 5d). The simulations show that increase in extracellular volume (and ECS permeability) changes PVS fluid entering the ECS, without affecting the fluid exchange between the PVS and the SAS, while the increased amplitude of vasodilation enhanced PVS fluid exchange with both SAS and ECS. If ECS permeability increased further, there was a further increase in the directional fluid flow from the PVS to the ECS. We varied the ECS permeability values over a range of physiologically plausible values [83, 103]. Specifically, the Darcy permeability ${k}_{s}^{2}$ varied from $5\times {10}^{-16}$ to $8\times {10}^{-15} {\text{m}}^{2}$, with corresponding values of hydraulic conductivity ${k}_{s}^{2}/{\mu }_{f}$ from $5\times {10}^{-13}$ to $8\times {10}^{-12} {\text{m}}^{2}/\left({\text{Pa}} \, {\text{s}}\right)$. Additional file 1: Figure. S5 shows that permeability is one of the most important parameters affecting fluid penetration into the ECS.

Our models also showed that the lower frequency arteriolar diameter changes that occur during sleep [111] could also play a major role in enhancing the directional fluid flow from the PVS to the ECS. We examined the role of frequency of arteriolar dilations in directional fluid flow by changing the dilation time (the time from the start of arteriolar dilation to the return to original size) in the asymmetric dilation waveform (Additional file 1: Fig. S6a) and using default values for all other parameters (see Table1). The models showed that amount of PVS fluid exchanged with the ECS increases almost linearly with the increase in dilation time (Additional file 1: Fig. S6b). Another way of thinking about the effect of sleep on vasodilation patterns is that the area under the dilation curve (area under curve for arteriolar wall displacement with time) increases during sleep. The effect of the area under the dilation curve on the convective fluid flow through the PVS is demonstrated by our simulations presented in the previous sections (Figs. 3, 4), which showed that for the same peak vasodilation amplitude, a temporally asymmetric waveform (with nearly two times the area under displacement curve as the symmetric waveform) can drive larger PVS fluid flow into the ECS compared to a symmetric waveform. To further investigate how vasodilation amplitude and area under dilation curve affect PVS fluid flow, we performed simulations with both asymmetric and symmetric dilation waveforms of different peak amplitudes (Additional file 1: Fig. S7). The simulations showed that for the same dilation amplitude, the directional fluid flow is appreciably affected by the waveform of the dilation (Additional file 1: Fig. S7a, b), while for the same area under dilation curve the directional fluid flow drive by arteriolar dilations is mostly unaffected by the dilation waveform (Additional file 1: Fig. S7c, d). Therefore, the larger area under dilation curve observed during sleep could also play an important role in directional fluid flow into the ECS through the PVS.

## Discussion

Here, we simulated fluid flow in a poroelastic model of the brain during the dilations of penetrating arterioles. We found that temporally asymmetric vasodilation drove directed fluid flow, with fluid flow from the PVS into the ECS during dilation, and fluid flows from SAS into the PVS during the return to baseline diameter. This could explain the importance of the speedup of vasodilation by noradrenergic stimulation [8], and how the slowing of vasodilation with age [43] could contribute to lower solute clearance from the brain. Moreover, given that the brain is oversupplied with oxygen, and that the oxygen changes during functional hyperemia exceed the increased oxygen demand due to neural activity [67, 128], it is possible that driving fluid movement through the PVS is one of the physiological purposes of functional hyperemia. Our poroelastic models showed that the shape, size and frequency of vasodilation, along with the permeability of the ECS are important factors that influence the amount of directional fluid flow from the PVS to the ECS. Based on the results of our simulations, the increased solute transport in the brain during sleep could be attributed to the increased ECS volume, along the large-amplitude, low-frequency vasodilation observed during sleep. While the dilation of arterioles associated with “fidgeting” motions [28, 30] and exercise [40, 41] will help clear waste in the awake brain, it will not be as effective as the larger dilations and porosity changes that occur during sleep.

The Reynold’s number and the spatial average flow velocities through the PVS in Additional file 1: Fig. S8 show that the CSF flow velocities are in the range of laminar flow. The peak flow velocities in the PVS of $30\, \upmu {\text{m}}/{\text{s}}$ for the awake state simulations are in the range of PVS velocities observed by in-vivo measurements [7, 78, 86]. The peak fluid velocities in the ECS in our model are in the order of $1$ to $10\, \upmu {\text{m}}/{\text{s}}$. Compared to the ECS fluid velocities of approximately $0.01$ to $0.1\, \upmu {\text{m}}/{\text{s}}$ predicted by previous studies [47, 50, 89], our models predict a higher ECS fluid velocity. This difference might be because the other models did not consider large dilations of arterioles observed during functional hyperemia. The vasodilation drives tissue deformation along with changes in fluid pressures which might drive larger fluid flow in the ECS.

Several studies have suggested that heartbeat-driven pulsations of arteries and arterioles can cause directional fluid flow through the PVS of arterioles [7, 23, 56, 78]. It is possible that heartbeat-driven pulsations, which have a temporally asymmetric waveform [34, 78], can cause directional fluid flow through the PVS of penetrating arterioles. However, issues concerning the choice of boundary conditions and the computational cost of the simulations need to be properly addressed to simulate the fluid flow driven by heartbeat pulsations in a poroelastic brain. While displacement boundary conditions are apt for simulating functional hyperemia, which is a large and active motion of smooth muscle cells and can occur even in brain slices with no perfusion pressure in arteries [31, 33, 68], it is unclear if heartbeat pulsations should be simulated by pressure or displacement boundary conditions, because pulsations are a direct result of pressure changes in the arteriolar lumen. The small scale of the heartbeat pulsations [7, 78, 86] also makes it hard to tease out the effect of pressure and displacement of the arterial wall. The choice of pressure/displacement boundary conditions could result in widely different predictions in our models, which showed that poroelastic mixtures have very distinct flow characteristics when subjected to pressure and displacement boundary conditions (Additional file 1: Fig. S5). The boundary conditions at the end of the SAS in the model also need to be reconsidered. The PVS of arterioles in the cerebral cortex is connected to the PVS of larger arteries, which include major branches of the middle cerebral artery [7, 78, 81] that also pulsate at heartrate. To understand fluid flow in the PVS of penetrating arterioles, the model needs to include coupled fluid chambers representing the fluid flow in the PVS of large arteries, at the SAS. Another important concern for accurately modeling pulsation-driven fluid flow is the computational cost of simulations. To simulate the frequency response of the model subject to heartbeat pulsations, we need to achieve a state where the change in variables from cycle to cycle is minimal. Since all our simulations have an initial condition where all the variables are set to zero, we need to simulate several cycles of pulsation to achieve the frequency response. In our fluid dynamic [62] and fluid–structure interaction [61] models, the frequency response was achieved by slowly ramping up the pulsation amplitude and simulating 20 cycles of pulsation, after which the cycle-to-cycle change in variables was less than 0.1%. Simulating 20 heartbeat cycles with our current 3D poroelastic models, which used a direct solver with 1.3 million unknowns, would be prohibitively expensive for our computational architecture. There are several factors that can affect peristalsis-driven fluid motion in the PVS surrounded by a poroelastic brain tissue like the length and curvature of the artery, the permeability of the brain tissue and the amplitude of the dilation [90]. The large number of variables affecting peristaltic flow suggest that a more computationally efficient model than the one presented here would be better suited to study pulsation-driven flow.

There are several ways that the model could be improved. The problem with the computational cost of the model could be addressed by implementing a stabilization techniques [75, 76, 85] for the incompressible poroelastic model, which would allow the usage of first-order interpolations for displacements and velocities, thereby reducing the number of unknowns. A more realistic geometry of the PVS could be used in the model to understand how factors like eccentricity of the PVS affect fluid flow [107]. Diffusion equations can be added to the model to simulate tracer infusion experiments more faithfully by including the physics of diffusion and altered mobility of solutes in porous fluid spaces [105]. Reduced-order models of the geometry simulated in this study could be used to simulate larger regions of the cortex to better understand the factors influencing large variations in CSF flow observed during exercise [49] and sleep [35, 122].

Our results could shed a new light on some aspects of the glymphatic hypothesis of solute transport in the brain. There has been controversy whether diffusion or convection dominates in the brain [54, 82]. Our models suggest that directional transport of into the brain is possible, but that it requires the active dilation and constriction of arteries to generate the movement. The model might also explain the controversy over glymphatic flow, in terms of the differences in solute transport in the brain depending on the anesthetic state [39]. Depending on anesthetic state, there may or may not be spontaneous arterial dilations, and without dynamic changes in arterial diameter, there will be much less convective fluid flow in the brain. For example, the lower solute transport seen under isoflurane anesthesia as compared to ketamine/xylazine anesthesia [42] could be explained in part by the fact that isoflurane is a strong vasodilator which can occlude vasodilation events [64]. Our models could also explain the role of Aqp4 in driving solute transport through the PVS. Knockouts of Aqp4 and α-Syntrophin genes could result in lower permeability at the PVS–ECS interface [37, 48], which could contribute the slower solute transport observed in the brains of Aqp4 and α-Syntrophin knockout mice [54, 77], although the effect of Aqp4 on the permeability at the PVS–ECS interfaces might be complicated and [59, 115] needs further investigation.

In contrast to the fluid pathway from the arteriolar PVS to the venular PVS proposed by the glymphatic hypothesis, our model suggests a pathway of fluid circulation, into the ECS through the PVS of arterioles and out through the surface of the brain. Although the model does not simulate the PVS of venules, the high flow resistance of the ECS and the small size of the PVS of venules compared to arteriolar PVS [114] suggest that the path of least resistance for fluid flow out of the ECS is through the brain surface, as predicted by our models, rather than through a long section of the ECS and then through the PVS of venules. This pathway of fluid circulation would explain why dyes injected into the CSF take longer to clear from the PVS of venules [54], where the fluid movement would be minimal, as veins do not dilate in anesthetized animals [29, 46]. However, we only simulated a small segment of arterioles penetrating into the brain. The path of least flow resistance for deeper sections of the PVS might be different from what is simulated in this study.

## Methods

### Model geometry

The geometry was created using Autodesk Inventor 2020 (San Rafael, Ca.). The two domains (see Fig. 2a), one representing the fluid-filled spaces (SAS and PVS) and one representing the brain parenchyma, were created as separate parts. An assembly was created by matching the two parts, and the assembly was exported into a standard exchange format (.step file), so that it can be accessed by any 3D CAD and meshing software. The step files are available on GitHub (https://github.com/kraviteja89/poroelastic3DPVS).

For the portion representing the fluid-filled spaces, the segment representing the PVS was created by using the loft function between two annular sections $150\, \upmu {\text{m}}$ apart along the $z$-axis. The cross section representing the SAS was created at $y=-100\,\upmu\mathrm{m}$ and extruded to $y=100\,\upmu\mathrm{m}$. The outer surface of the intersection between the two solids was smoothened by using a fillet of radius $7\, \upmu {\text{m}}$. The cavity representing the arteriole passing through the SAS was created by using the sweep function on a circular face along a path including a straight line and an arc to connect to the surface and penetrating segments of the arteriole. The solid was split at the $yz$-plane ($x=0$).

For the part representing the brain tissue, a block of size $160\, \upmu {\text{m}}\times 200\, \upmu {\text{m}}\times 170\, \upmu {\text{m}}$ was created by extruding a rectangular face. A cut was made by extruding a negative volume based on the bottom face of the SAS. Another cut was made by using the loft function on two circles representing the outer wall of the PVS. A fillet was made at the intersection of the two faces. The solid was split at the $yz$-plane ($x=0$).

### Meshing

A custom tetrahedral mesh was generated for the geometry using Altair Hypermesh. The mesh is shown in Fig. 2b. A hexahedral mesh was first created for the PVS surrounding the penetrating segment of the arteriole, with 4 elements along the width of the PVS, 16 elements along the half circumference, and an element height of $3\, \upmu {\text{m}}$ near the surface and $6\, \upmu {\text{m}}$ at $150\, \upmu {\text{m}}$ below the surface. Two layers of hexahedral elements of width $1.5$ and $2.5\, \upmu {\text{m}}$ perpendicular to the surfaces were created at the interface between the two parts of the geometry, the arteriolar wall in the SAS and the top surface of the dura ($z=200\, \upmu {\text{m}}$). The hexahedral elements were created to control the mesh shape at the interface between the two parts of the geometry and the boundaries where no-slip boundary conditions were applied. These hexahedrons were split into tetrahedrons and controlled triangular meshes were created on the remaining surfaces of each part. The triangular faces of the existing tetrahedrons, along with the triangular meshes on the remining surfaces were used to generate a tetrahedral volume meshes. Quality of mesh was maintained by setting a minimum tet collapse ($1.24\times{}$ ratio of distance between a node from the opposite triangular face to the area of the face) of $0.15$. The mesh was exported into the Nastran format (.nas), which was imported into COMSOL Multiphysics.

### Model formulation

A poroelastic model [20] was used to simulate fluid flow through the SAS, PVS and the ECS, along with the deformations of the connective and parenchymal tissue. The model was divided into two domains, one representing the fluid-filled PVS and SAS (${\Omega }^{1}$) and the other representing the parenchymal tissue (${\Omega }^{2}$), as described in the model geometry section. In each domain, we solve for five unknowns ${{\varvec{u}}}_{s}^{a}, {{\varvec{v}}}_{s}^{a}, {{\varvec{v}}}_{f}^{a}, {{\varvec{v}}}_{flt}^{a}$ and ${p}^{a}$ (4 vectors and 1 scalar) representing the solid displacement, solid velocity, fluid velocity, filtration velocity and pore pressure respectively. The superscript, $a=1, 2$ represents the domain. In both domains, an arbitrary Lagrangian–Eulerian (ALE) finite element formulation of poroelasticity, based on mixture theory was implemented to simulate an incompressible hyperelastic skeleton saturated with an incompressible fluid. The development of the formulation is explained in detail by Costanzo and Miller [20]. The key equations of the formulation are described below.

### Kinematics

The ALE formulation was written in the coordinates of the undeformed solid skeleton (${{\varvec{X}}}_{s}^{a}$). The domains in the reference (undeformed solid) frame are represented by ${\widehat{\Omega }}^{1}$ and ${\widehat{\Omega }}^{2}$. If the deformed configuration is given by ${{\varvec{x}}}_{s}^{a}(= {{\varvec{\chi}}}_{s}^{a}\left({{\varvec{X}}}_{s}^{a}\right),$ where ${{\varvec{\chi}}}_{s}^{a}$ is a smooth map describing the transformation from the deformed state to the reference configuration), the displacement (${{\varvec{u}}}_{s}^{a}$), deformation gradient (${{\varvec{F}}}_{s}^{a}$) and Jacobian determinant (${J}_{s}^{a}$) of the motion are given by Eq. (1). In Eq. (1), $\nabla$ is the gradient operator with respect to the reference coordinates, ${\varvec{I}}$ is the identity tensor, and $\mathrm{det}$ is the determinant.

1 $${{\varvec{u}}}_{s}^{a}\left({{\varvec{X}}}_{s}^{a}, t\right) := {{\varvec{\chi}}}_{s}^{a}\left({{\varvec{X}}}_{s}^{a}\right)- {{\varvec{X}}}_{s}^{a}, {{\varvec{F}}}_{s}^{a}\left({{\varvec{X}}}_{s}^{a}, t\right) := \frac{\partial {{\varvec{\chi}}}_{s}^{a}}{\partial {{\varvec{X}}}_{s}^{a}}=\nabla {{\varvec{u}}}_{s}^{a}+{\varvec{I}}, \quad {J}_{s}^{a}\left({{\varvec{X}}}_{s}^{a}, t\right)=\mathrm{det}{{\varvec{F}}}_{s}^{a}.$$

Since the equations are written in the material particle coordinates of the solid skeleton, the relation between solid displacement and solid velocity is given by Eq. (2).

2 $${{\varvec{v}}}_{s}^{a}\left({{\varvec{X}}}_{s}^{a}, t\right)= \frac{\partial {{\varvec{u}}}_{s}^{a}\left({{\varvec{X}}}_{s}^{a}, t\right)}{\partial t}.$$

At each point, the volume fraction of the solid and fluid are given by ${\zeta }_{s}^{a}$ and ${\zeta }_{f}^{a}$, respectively and the mass densities are given by ${\rho }_{s}^{a}$ and ${\rho }_{f}^{a}$, respectively. The mass densities are related to the true densities (${\rho }_{s}^{*}$ and ${\rho }_{f}^{*}$) of the solid and fluid phases by Eq. (3). The true mass density of a component of the mixture is defined as the mass density of that component in its single-phase state. Note that the true densities are constants for incompressible phases and hence the superscript is omitted. Since the solid is fully saturated by the fluid, the sum of their volume fractions is unity, which is the constraint shown in Eq. (4).

3 $$\rho_{s}^{a}= {\zeta }_{s}^{a} {\rho }_{s}^{*},\hspace{0.33em}{\rho }_{f}^{a}= {\zeta }_{f}^{a} {\rho }_{f}^{*}.$$

4 $${\zeta }_{s}^{a}+ {\zeta }_{f}^{a}=1.$$

As the solid deforms, the volume fractions of the phases evolve continuously. The relation between, ${\zeta }_{{R}_{s}}^{a}$, the volume fraction of the solid in the undeformed reference configuration and the actual volume fractions of the phases are given by Eq. (5).

5 $${\zeta }_{s}^{a}= \frac{{\zeta }_{{R}_{s}}^{a}}{{J}_{s}^{a}}, {\zeta }_{f}^{a}= 1- \frac{{\zeta }_{{R}_{s}}^{a}}{{J}_{s}^{a}}.$$

The filtration velocity ${{\varvec{v}}}_{flt}^{a}$ is defined as the velocity of the fluid relative to the solid skeleton scaled by the volume fraction of the fluid, as shown in Eq. (6).

6 $${{\varvec{v}}}_{flt}^{a}= {\zeta }_{f}^{a}\left({{\varvec{v}}}_{f}^{a}- {{\varvec{v}}}_{s}^{a}\right)= \left(1- \frac{{\zeta }_{{R}_{s}}^{a}}{{J}_{s}^{a}}\right)\left({{\varvec{v}}}_{f}^{a}- {{\varvec{v}}}_{s}^{a}\right).$$

In the absence of chemical reactions, the incompressibility constraint is given by the zero divergence of the volume averaged velocity, thus yielding the constraint shown in Eq. (7), where ${\text{div}}$ and ${\text{grad}}$ are the divergence and gradients with respect to the deformed coordinates${{\varvec{x}}}_{s}^{a}$, while the colon $:$ denotes the inner product of tensors. Using the chain rule and Eq. (6), the incompressibility constraint, written in terms of quantities defined in the ALE coordinates, takes the form shown in Eq. (8), where ${{\varvec{A}}}^{-1}$ and ${{\varvec{A}}}^{T}$ are the inverse and transpose operations respectively on the tensor${\varvec{A}}$:

7 $$0={\text{div}}\left({\zeta }_{s}^{a}{{\varvec{v}}}_{s}^{a}+{\zeta }_{f}^{a}{{\varvec{v}}}_{f}^{a}\right)={\varvec{I}}:{\text{grad}}\left({\zeta }_{s}^{a}{{\varvec{v}}}_{s}^{a}+{\zeta }_{f}^{a}{{\varvec{v}}}_{f}^{a}\right).$$

8 $$0= {\varvec{I}}:{{{\varvec{F}}}_{s}^{a}}^{-1}\nabla \left({{\varvec{v}}}_{s}^{a}+ {{\varvec{v}}}_{flt}^{a}\right)= {{{\varvec{F}}}_{s}^{a}}^{-T}:\nabla \left({{\varvec{v}}}_{s}^{a}+ {{\varvec{v}}}_{flt}^{a}\right).$$

### Constitutive assumptions and momentum balance

The solid skeleton is modeled as an isotropic incompressible neo-Hookean solid and the fluid flow is modeled by incompressible Darcy-Brinkman law for flow through porous solids. The total Cauchy stress of the mixture is given by Eq. (9), where, ${{\varvec{\sigma}}}_{s}^{a}$ is the elastic contribution due to the strain energy density (${\Psi }_{s}$), while ${{\varvec{\sigma}}}_{f}^{a}$ accounts for the Brinkman dissipation. In Eq. (10), the shear modulus of the solid skeleton for domain $a$ is given by ${\mu }_{s}^{a}$. The strain energy density in Eq. (10) appears similar to that of a compressible solid, which is a valid choice of constitutive model because even though the pure solid constituent is incompressible, the solid skeleton of the porous solid can be compressed [87, 108] and it has the convenience of yielding the expression ${{\varvec{\sigma}}}_{s}^{a}=0$ in the reference configuration. In Eq. (11) ${\mu }_{f}$ is the dynamic viscosity of the fluid. It is important to note that the stresses in Eqs. (9)–(11) are defined in terms of the deformed coordinates.

9 $${{\varvec{\sigma}}}^{a}= -{p}^{a}{\varvec{I}}+ {{\varvec{\sigma}}}_{s}^{a}+{{\varvec{\sigma}}}_{f}^{a}.$$

10 $${{\varvec{\sigma}}}_{s}^{a}=\frac{2}{{J}_{s}^{a}}{{\varvec{F}}}_{s}^{a}\frac{\partial {\Psi }_{s}}{\partial {{\varvec{C}}}_{s}^{a}}{{{\varvec{F}}}_{s}^{a}}^{T},{\Psi }_{s}=\frac{{\mu }_{s}^{a}}{2}\left(Tr\left[{{\varvec{C}}}_{s}^{a}\right]-2\mathrm{ln}{J}_{s}^{a}\right), {{\varvec{C}}}_{s}^{a}={{{\varvec{F}}}_{s}^{a}}^{T}{{\varvec{F}}}_{s}^{a}.$$

11 $${{\varvec{\upsigma}}}_{f}^{a}={\, \mu }_{f} \left({\text{grad}}{{\varvec{v}}}_{flt}^{a}+{\left({\text{grad}}{{\varvec{v}}}_{flt}^{a}\right)}^{T}\right).$$

The momentum equations can be written in the ALE coordinates based on the stresses defined in Eqs. (9–11) and the chain rule for transforming the spatial gradients. Equations (12) and (13) are the momentum equations for the solid and fluid components of the mixture, respectively. The definitions of ${{\varvec{P}}}_{s}^{a}$ and ${{\varvec{P}}}_{f}^{a}$ are given by Eqs. (14) and (15), respectively.

12 $$0= {\zeta }_{{R}_{s}}^{a}{\rho }_{s}^{*}\frac{\partial {{\varvec{v}}}_{s}^{a}}{\partial t}+ {\zeta }_{{R}_{s}}^{a}{{{\varvec{F}}}_{s}^{a}}^{-T}\nabla {p}^{a}-\left({J}_{s}^{a}- {\zeta }_{{R}_{s}}^{a}\right)\frac{{\mu }_{f}}{{k}_{s}^{a}}{{\varvec{v}}}_{flt}^{a}- \nabla \!\cdot\!{{\varvec{P}}}_{s}^{a}.$$

13 $$0=\left({J}_{s}^{a}- {\zeta }_{{R}_{s}}^{a}\right)\left({\rho }_{f}^{*}\frac{\partial {{\varvec{v}}}_{f}^{a}}{\partial t}+\frac{{J}_{s}^{a}{\rho }_{f}^{*}}{\left({J}_{s}^{a}- {\zeta }_{{R}_{s}}^{a}\right)}{{{\varvec{F}}}_{s}^{a}}^{-1}\left(\nabla {{\varvec{v}}}_{f}^{a}\right){{\varvec{v}}}_{flt}^{a}+{{{\varvec{F}}}_{s}^{a}}^{-T}\nabla {p}^{a}+\frac{{\mu }_{f}}{{k}_{s}^{a}}{{\varvec{v}}}_{flt}^{a}\right)-\nabla\!\cdot\!{{\varvec{P}}}_{f}^{a}.$$

14 $${{\varvec{P}}}_{s}^{a} = {\mu }_{s}^{a} \left({{\varvec{F}}}_{s}^{a}- {{{\varvec{F}}}_{s}^{a}}^{-T}\right).$$

15 $${{\varvec{P}}}_{f}^{a}= {\mu }_{f}\left({J}_{s}^{a}- {\zeta }_{{R}_{s}}^{a}\right)\left({\nabla {\varvec{v}}}_{flt}^{a}{{{\varvec{F}}}_{s}^{a}}^{-1}+ {\left({\nabla {\varvec{v}}}_{flt}^{a}{{{\varvec{F}}}_{s}^{a}}^{-1}\right)}^{T}\right).$$

Within each domain, we have five equations, Eqs. (2), (6), (8), (12), and (13), that govern the spatiotemporal evolution of the five primary unknowns.

### Interface conditions

At the interface of the mostly fluid-filled spaces i.e., the SAS and the PVS with the brain parenchyma, there is a sharp change (mathematically, a possible jump discontinuity) in the volume fraction of the fluid (porosity) and the composition of the solid skeleton. Therefore, there is a sharp change in the fluid permeability and elastic modulus which could result in a sharp change in the fluid velocity and the distribution of traction between the solid and fluid components at the interface. To deal with the sharp change, the mass and traction continuity at the interface were implemented through special boundary conditions called jump conditions [24, 98, 99].

The solid phases in both the domains are in contact with each other at the interface and therefore the solid displacement and the velocity are continuous across the interface, as indicated in Eq. (17). For an incompressible fluid (fluid with constant true density), the mass conservation for the fluid across the interface dictates that the component of the filtration velocity normal to the interface should be continuous across the boundary. By considering the limiting case as $${\zeta }_{s}^{1}\to 0$$, Hou et al. [52] showed that the no-slip condition for both extremes at the other end, $$\text{${\zeta }_{s}^{1}\to 0$ and ${\zeta }_{s}^{1}\to 1$}$$) are valid when the tangential component of filtration velocity is continuous across the interface. The continuity of both tangential and normal components of the filtration velocities implies the continuity of filtration velocity across the interface, indicated in Eq. (17):

16 $${{\varvec{u}}}_{s}^{1}= {{\varvec{u}}}_{s}^{2}, \quad {{\varvec{v}}}_{s}^{1}= {{\varvec{v}}}_{s}^{2}.$$

17 $${{\varvec{v}}}_{flt}^{1}= {{\varvec{v}}}_{flt}^{2}.$$

Equation (18) states the condition that the *total* traction force across the interface be continuous. In Eq. (18), ${{\varvec{n}}}^{1}$ and ${{\varvec{n}}}^{2}$ are the unit outward normal to the domains ${\Omega }^{1}$ and ${\Omega }^{2}$, respectively. Additionally, we assume that the ratio of tractions on each phase is equal to the ratio of the volume fractions of the phase. This assumption is stated in Eqs. (19) and (20).

18 $${{\varvec{\sigma}}}^{2}{{\varvec{n}}}^{2}= {{\varvec{\sigma}}}^{1}{{\varvec{n}}}^{1}.$$

19 $$\left({-{{\zeta}}_{s}^{2}{p}^{2}{\varvec{I}}\boldsymbol{ }+\boldsymbol{ }{\varvec{\sigma}}}_{s}^{2}\right){{\varvec{n}}}^{2}= {{\zeta}}_{s}^{2}{{\varvec{\sigma}}}^{2}{{\varvec{n}}}^{2}= {{\zeta}}_{s}^{2}{{\varvec{\sigma}}}^{1}{{\varvec{n}}}^{1}= {{\zeta}}_{s}^{2}\left({-{p}^{1}{\varvec{I}}\boldsymbol{ }+\boldsymbol{ }{\varvec{\sigma}}}_{s}^{1}{\boldsymbol{ }+\boldsymbol{ }{\varvec{\sigma}}}_{f}^{1}\right){{\varvec{n}}}^{1}.$$

20 $$\left({-{{\zeta}}_{f}^{2}{p}^{2}{\varvec{I}}\boldsymbol{ }+\boldsymbol{ }{\varvec{\sigma}}}_{f}^{2}\right){{\varvec{n}}}^{2}= {{\zeta}}_{f}^{2}\left({-{p}^{1}{\varvec{I}}\boldsymbol{ }+\boldsymbol{ }{\varvec{\sigma}}}_{s}^{1}{\boldsymbol{ }+\boldsymbol{ }{\varvec{\sigma}}}_{f}^{1}\right){{\varvec{n}}}^{1}.$$

Equations (18)–(20) are written in the deformed configuration. The unit outward normal to ${\Omega }^{a}$, ${{\varvec{n}}}^{a}$, is related to the unit outward normal to ${\widehat{\Omega }}^{a}$ (the undeformed domain), ${\widehat{{\varvec{n}}}}^{a}$, according to the relation in Eq. (21). Using Eqs. (14), (15), and (21), the traction conditions at the interface can then be rewritten in ALE coordinates as shown in Eqs. (22) and (23).

21 $${{\varvec{n}}}^{a}= {J}_{s}^{a}{{{\varvec{F}}}_{s}^{a}}^{-T}{\widehat{{\varvec{n}}}}^{a}.$$

22 $$\left({-{{\zeta}}_{{R}_{s}}^{2}{p}^{2}{{{\varvec{F}}}_{s}^{2}}^{-T}\boldsymbol{ }+\boldsymbol{ }{\varvec{P}}}_{s}^{2}\right){\widehat{{\varvec{n}}}}^{2}= \frac{{{\zeta}}_{{R}_{s}}^{2}}{{J}_{s}^{2}}{{\varvec{P}}}_{mix}^{1}{\widehat{{\varvec{n}}}}^{1}= \frac{{{\zeta}}_{{R}_{s}}^{2}}{{J}_{s}^{2}}\left({-{J}_{s}^{1}{p}^{1}{{{\varvec{F}}}_{s}^{1}}^{-T}\boldsymbol{ }+\boldsymbol{ }{\varvec{P}}}_{s}^{1}+ {{\varvec{P}}}_{f}^{1}\right){\widehat{{\varvec{n}}}}^{1}.$$

23 $$\left({-\left({{J}_{s}^{2}-\boldsymbol{ }{\zeta}}_{{R}_{s}}^{2}\right){ p}^{2}{{{\varvec{F}}}_{s}^{2}}^{-T}\boldsymbol{ }+\boldsymbol{ }{\varvec{P}}}_{f}^{2}\right){\widehat{{\varvec{n}}}}^{2}= \frac{{{J}_{s}^{2}-\boldsymbol{ }{\zeta}}_{{R}_{s}}^{2}}{{J}_{s}^{2}}{{\varvec{P}}}_{mix}^{1}{\widehat{{\varvec{n}}}}^{1}.$$

### Boundary conditions

For the segment of the arteriole in the SAS ($z>150\, \upmu {\text{m}}$), the displacement is prescribed against the direction of the outward normal (Eq. 24). For the segment of the arteriole ($z\le 150\, \upmu {\text{m}}$), the displacement is prescribed along the radially outward direction as shown in Eq. (25) where ${R}_{0}$ is the nominal radius of the vessel (see Table 1). The solid velocity on the arteriolar wall is the partial time derivative of the prescribed displacement, shown in Eq. (26), where ${{\varvec{u}}}_{s0}^{1}$ is the displacement prescribed by Eqs. (24) and (25). The no-slip condition is implemented by setting the filtration velocity to zero.

24 $${\text {On the arteriolar wall}}, \quad {{\varvec{u}}}_{{s}^{1}}=- \mathrm{an}1 {\widehat{{\varvec{n}}}}^{1}\hspace{0.33em}\hspace{0.33em} \quad \text{ for} \; \hspace{0.25em} z>150\, \upmu {\text{m}},$$

25 $${u}_{sx}^{1}=\frac{x}{{R}_{0}}an1, {\boldsymbol{ }\boldsymbol{ }\boldsymbol{ }u}_{sy}^{1}=\frac{y}{{R}_{0}}an1, { u}_{sz}^{1}=0 \quad for\;z\le 150.$$

26 $${{\varvec{v}}}_{s}^{1}= \frac{\partial {{\varvec{u}}}_{s0}^{1}}{\partial t}.$$

27 $${{\varvec{v}}}_{flt}^{1}=0.$$

At the bottom face of the fluid-filled domain (${\widehat{\Omega }}^{1}$), the solid displacement and velocity in the $z$ direction were set to zero (Eq. 28). On the fluid phase, a flow-dependent traction (flow resistance) boundary condition was used. The flow resistance at the bottom end of the PVS was set to 10 times the resistance of an annular region with the permeability of the PVS, inner radius of $7.5\, \upmu {\text{m}}$ (${R}_{2}$) and a width of $5.5\, \upmu {\text{m}}$ (${W}_{2}$). In Eq. (30), ${L}_{a}$ is the height of the PVS segment ($150\, \upmu {\text{m}}$) and ${Q}_{1}$ is the flowrate through the bottom face calculated by the integral over the bottom face ($\partial {\widehat{\Omega }}^{1}$).

28 $$\text{At}\,\, z=0, { u}_{sz}^{1}=0, { v}_{sz}^{1}=0.$$

29 $$\left({-\left({{J}_{s}^{1}-\boldsymbol{ }{\zeta}}_{{R}_{s}}^{1}\right){ p}^{1}{{{\varvec{F}}}_{s}^{1}}^{-T}\boldsymbol{ }+\boldsymbol{ }{\varvec{P}}}_{f}^{1}\right){\widehat{{\varvec{n}}}}^{1}= -\left({{J}_{s}^{1}-\boldsymbol{ }{\zeta}}_{{R}_{s}}^{1}\right){p}_{Robin}{{{\varvec{F}}}_{s}^{1}}^{-T}{\widehat{{\varvec{n}}}}^{1}.$$

30 $${p}_{Robin}= 10 \frac{{L}_{a}{\mu }_{f}}{{k}_{s}} \frac{{Q}_{1}}{\pi \left({\left({R}_{2}+ {W}_{2}\right)}^{2}- {{R}_{2}}^{2}\right)}, {Q}_{1}= \underset{\partial {\widehat{\Omega }}^{1}}{\overset{}{\int }}{J}_{s}^{1}{{\varvec{v}}}_{flt}^{1}\cdot {{{{\varvec{F}}}_{s}^{1}}^{-T}\widehat{{\varvec{n}}}}^{1}.$$

The circulation of CSF in the SAS was simulated by applying a small pressure difference across the ends of the SAS on the fluid component (green and blue faces in Fig. 2a). The solid displacement and velocity in the *y* directions were set to zero at the ends of the SAS.

31 $$\text{At}\,\, y=-100, \left({-\left({{J}_{s}^{1}-\boldsymbol{ }{\zeta}}_{{R}_{s}}^{1}\right){ p}^{1}{{{\varvec{F}}}_{s}^{1}}^{-T}\boldsymbol{ }+\boldsymbol{ }{\varvec{P}}}_{f}^{1}\right){\widehat{{\varvec{n}}}}^{1}= -\left({{J}_{s}^{1}-\boldsymbol{ }{\zeta}}_{{R}_{s}}^{1}\right){p}_{0}{{{\varvec{F}}}_{s}^{1}}^{-T}{\widehat{{\varvec{n}}}}^{1}.$$

32 $$\text{At}\,\, y=100, \left({-\left({{J}_{s}^{1}-\boldsymbol{ }{\varvec{\zeta}}}_{{R}_{s}}^{1}\right){ p}^{1}{{{\varvec{F}}}_{s}^{1}}^{-T}\boldsymbol{ }+\boldsymbol{ }{\varvec{P}}}_{f}^{1}\right){\widehat{{\varvec{n}}}}^{1}= 0.$$

33 $$\text{At}\,\,y=-100, \, and \, y=100 \quad { u}_{sy}^{1}=0, { v}_{sy}^{1}=0.$$

At the top surface of the SAS, which represents the arachnoid bounded by the dura, all the velocities and displacements were set to zero.

34 $$\text{At}\,\,z=200, \quad { {\varvec{u}}}_{s}^{1}=0, \quad { {\varvec{v}}}_{s}^{1}=0, \quad { {\varvec{v}}}_{flt}^{1}=0.$$

At $x=0$ and $x=80$, symmetry boundary conditions were used, where the solid displacement, velocity and filtration velocity normal to the surface were set to zero.

35 $$\text{On} \; \partial {{\widehat{\Omega }}^{1}}_{s}, \quad {{\varvec{u}}}_{s}^{1}\cdot {\widehat{{\varvec{n}}}}^{1}=0, \quad {{\varvec{v}}}_{s}^{1}\cdot {\widehat{{\varvec{n}}}}^{1}=0, \quad {{\varvec{v}}}_{flt}^{1}\cdot {\widehat{{\varvec{n}}}}^{1}=0.$$

At all the surfaces on the domain representing the brain tissue (${\widehat{\Omega }}^{2}$), other than the interface with the fluid-filled domain, the normal components of displacements and velocities were set to zero. At the plane of symmetry ($x=0$), the boundary condition is self-explanatory. The use of the boundary condition at the side-facing surfaces ($y= -100$, $y= 100$ and $x= 80$), the boundary condition represents the assumption that the region being modeled is surrounded by similar blocks of the brain tissue experiencing vasodilation. On the bottom surface, a flow resistance boundary condition, similar to the one presented in Eq. (29) was not applied, as it would set a constant traction force on the whole surface.

36 $$\text{On} \; \partial {{\widehat{\Omega }}^{2}}_{s}, \quad {{\varvec{u}}}_{s}^{2}\cdot {\widehat{{\varvec{n}}}}^{2}=0, \quad {{\varvec{v}}}_{s}^{2}\cdot {\widehat{{\varvec{n}}}}^{2}=0, \quad {{\varvec{v}}}_{flt}^{2}\cdot {\widehat{{\varvec{n}}}}^{2}=0$$

### Finite element implementation

The overall initial-boundary value problem formulated in the previous section is complex and nonlinear. To the authors’ knowledge, there are no formal proofs available in the literature to guide us in the formulation of a well-posed weak problem with minimum smoothness requirements for the various unknown fields under reasonable smoothness assumptions on the problem’s data. Consequently, here we make assumptions as to the functional spaces that we wish to have available to make the writing of the problem meaningful. Our numerical results indicate that our finite element implementation of the problem is adequate [20]. However, we wish to be clear that we are in no position to offer rigorous proofs as yet on the well-posedness of the problem. First, we consider vector functional spaces ${\mathcal{V}}^{1}$ and${\mathcal{V}}^{2}$, along with scalar functional spaces ${\mathcal{P}}^{1}$ and $, {\mathcal{P}}^{2}$ that are defined as follows.

37 $${\mathcal{V}}^{1} := \left\{{\varvec{u}} \in {L}^{2}{\left({\widehat{\Omega }}^{1}\right)}^{3}| \nabla {\varvec{u}} \in {L}^{2}{\left({\widehat{\Omega }}^{1}\right)}^{3X3}\right\}.$$

38 $${\mathcal{V}}^{2} := \left\{{\varvec{u}} \in {L}^{2}{\left({\widehat{\Omega }}^{2}\right)}^{3}| \nabla {\varvec{u}} \in {L}^{2}{\left({\widehat{\Omega }}^{2}\right)}^{3X3}\right\}.$$

39 $${\mathcal{P}}^{1} := \left\{p \in {L}^{2}\left({\widehat{\Omega }}^{1}\right)| \nabla p \in {L}^{2}{\left({\widehat{\Omega }}^{1}\right)}^{3}\right\}.$$

40 $${\mathcal{P}}^{2} := \left\{p \in {L}^{2}\left({\widehat{\Omega }}^{2}\right)| \nabla p \in {L}^{2}{\left({\widehat{\Omega }}^{2}\right)}^{3}\right\}.$$

Next, we define the solution spaces for ${{\varvec{u}}}_{s}^{1}$, ${{\varvec{v}}}_{s}^{1}$, ${{\varvec{v}}}_{flt}^{1}$ as subsets of ${\mathcal{V}}^{1}$. The boundaries of the domain ${\widehat{\Omega }}^{1}$ are divided into four subsets such that, ${\partial \widehat{\Omega }}^{1}= \partial {{\widehat{\Omega }}^{1}}_{D}\cup$ $\partial {{\widehat{\Omega }}^{1}}_{S} \cup$ $\partial {{\widehat{\Omega }}^{1}}_{I}$ $\cup$ $\partial {{\widehat{\Omega }}^{1}}_{N}$, where $\partial {{\widehat{\Omega }}^{1}}_{D}$, $\partial {{\widehat{\Omega }}^{1}}_{S}$, $\partial {{\widehat{\Omega }}^{1}}_{N}$ are the surfaces where Dirichlet (vessel wall and skull), symmetry ($x=0$ and $x=80$) and Neumann ($y=-100$, $y=100$ and $z=0$) boundary conditions are prescribed on the fluid phase, while $\partial {{\widehat{\Omega }}^{1}}_{I}$ is the interface boundary between the two domains.

41 $${\mathcal{V}}_{{{\varvec{u}}}_{s}}^{1}:=\left\{{{\varvec{u}}}_{s} \in {\mathcal{V}}^{1} , {{\varvec{u}}}_{s}={\overline{{\varvec{u}}} }_{s} \,\text{on}\, \partial {{\widehat{\Omega }}^{1}}_{D}, {{\varvec{u}}}_{s}\cdot {\widehat{{\varvec{n}}}}^{1}=0\,\text{on}\, \partial {{\widehat{\Omega }}^{1}}_{s} \cup \partial {{\widehat{\Omega }}^{1}}_{N} , {{\varvec{u}}}_{s}={{\varvec{u}}}_{s}^{2} \,\text{on}\, \partial {{\widehat{\Omega }}^{1}}_{I}\right\}.$$

42 $${\mathcal{V}}_{{{\varvec{v}}}_{s}}^{1} :=\left\{{{\varvec{v}}}_{s} \in {\mathcal{V}}^{1} , {{\varvec{v}}}_{s}={\overline{{\varvec{v}}} }_{s} \,\text{on}\, \partial {{\widehat{\Omega }}^{1}}_{D}, {{\varvec{v}}}_{s}\cdot {\widehat{{\varvec{n}}}}^{1}=0 \,\text{on}\, \partial {{\widehat{\Omega }}^{1}}_{s} \cup \partial {{\widehat{\Omega }}^{1}}_{N} , {{\varvec{v}}}_{s}={{\varvec{v}}}_{s}^{2} \,\text{on}\, \partial {{\widehat{\Omega }}^{1}}_{I}\right\}.$$

43 $${\mathcal{V}}_{{{\varvec{v}}}_{flt}}^{1}:=\left\{{{\varvec{v}}}_{flt}\in {\mathcal{V}}^{1} , {{\varvec{v}}}_{flt}={\overline{{\varvec{v}}} }_{flt} \,\text{on}\, \partial {{\widehat{\Omega }}^{1}}_{D}, {{\varvec{v}}}_{flt}\cdot {\widehat{{\varvec{n}}}}^{1}=0 \,\text{on}\, \partial {{\widehat{\Omega }}^{1}}_{s} , {{\varvec{v}}}_{flt}={{\varvec{v}}}_{flt}^{2} \,\text{on}\, \partial {{\widehat{\Omega }}^{1}}_{I}\right\}.$$

We also define the companion spaces for the test functions as follows.

44 $${\stackrel{\sim }{\mathcal{V}}}_{s}^{1} :=\left\{{\varvec{u}} \in {\mathcal{V}}^{1} , {\varvec{u}}=0 \,\text{on}\, \partial {{\widehat{\Omega }}^{1}}_{D}\cup \partial {{\widehat{\Omega }}^{1}}_{I}, {\varvec{u}}\cdot {\widehat{{\varvec{n}}}}^{1}=0 \,\text{on}\, \partial {{\widehat{\Omega }}^{1}}_{s} \cup \partial {{\widehat{\Omega }}^{1}}_{N} \right\}.$$

45 $${\stackrel{\sim }{\mathcal{V}}}_{flt}^{1} :=\left\{{\varvec{v}} \in {\mathcal{V}}^{1} , {\varvec{v}}=0 \,\text{on}\, \partial {{\widehat{\Omega }}^{1}}_{D} \cup \partial {{\widehat{\Omega }}^{1}}_{I}, {\varvec{v}}\cdot {\widehat{{\varvec{n}}}}^{1}=0 \,\text{on}\, \partial {{\widehat{\Omega }}^{1}}_{s} \right\} .$$

The solutions and test functions for ${{\varvec{v}}}_{f}^{1}$ and ${p}^{1}$ are taken from ${\mathcal{V}}^{1}$ and ${\mathcal{P}}^{1}$, respectively.

For ${\widehat{\Omega }}^{2}$, the boundary is divided into two non-intersecting subsets, $\partial {{\widehat{\Omega }}^{2}}_{S}$ and $\partial {{\widehat{\Omega }}^{2}}_{I}$, representing the boundaries with the symmetry and interface conditions, respectively. The solution and test functions for ${{\varvec{u}}}_{s}^{2}$, ${{\varvec{v}}}_{s}^{2}$, ${{\varvec{v}}}_{flt}^{2}$ belong to the same functional space (${\mathcal{V}}_{u}^{2}$ in Eq. 46). The solutions and test functions for ${{\varvec{v}}}_{f}^{1}$ and ${p}^{2}$ are taken from ${\mathcal{V}}^{2}$ and ${\mathcal{P}}^{2}$, respectively.

46 $${\mathcal{V}}_{u}^{2} :=\left\{{\varvec{u}} \in {\mathcal{V}}^{2} , {\varvec{u}}\cdot {\widehat{{\varvec{n}}}}^{2}=0 \,\text{on}\, \partial {{\widehat{\Omega }}^{2}}_{s} \right\}.$$

To simplify the weak form, we define the following notation, where $a=1, 2$, represents the domain and $\partial {\widehat{\Omega }}^{a}$ represents the boundary.

47 $${\left({\varvec{u}}, {\varvec{w}}\right)}_{a} := {\int }_{{\widehat{\Omega }}^{a}}{\varvec{u}}\cdot {\varvec{w}},\,\, b{\left({\varvec{u}},{\varvec{\sigma}}\right)}_{a} := {\int }_{{\widehat{\Omega }}^{a}}\nabla {\varvec{u}}:{\varvec{\sigma}}, \,\,{\left({\varvec{u}}, {\varvec{w}}\right)}_{\partial a} := {\int }_{\partial {\widehat{\Omega }}^{a}}{\varvec{u}}\cdot {\varvec{w}}.$$

The weak form of the problem can be written as follows:

$$Find\, {{\varvec{u}}}_{s}^{1}\in {\mathcal{V}}_{{{\varvec{u}}}_{s}}^{1}, {{\varvec{v}}}_{s}^{1}\in {\mathcal{V}}_{{{\varvec{v}}}_{s}}^{1}, {{\varvec{v}}}_{flt}^{1}\in {\mathcal{V}}_{{{\varvec{v}}}_{flt}}^{1}, {{\varvec{v}}}_{f}^{1}\in {\mathcal{V}}^{1}, {p}^{1}\in {\mathcal{P}}^{1}, {{\varvec{u}}}_{s}^{2},{{\varvec{v}}}_{s}^{2},{{\varvec{v}}}_{flt}^{2}\in {\mathcal{V}}_{u}^{2}, {{\varvec{v}}}_{f}^{2} \in {\mathcal{V}}^{2}, {p}^{2} \in {\mathcal{P}}^{2},\, such\; that\,\forall {\stackrel{\sim }{{\varvec{u}}}}_{s}^{1}, {\stackrel{\sim }{{\varvec{v}}}}_{s}^{1}\in {\stackrel{\sim }{\mathcal{V}}}_{s}^{1}, {\stackrel{\sim }{{\varvec{v}}}}_{flt}^{1}\in {\stackrel{\sim }{\mathcal{V}}}_{flt}^{1}, {\stackrel{\sim }{{\varvec{v}}}}_{f}^{1}\in {\mathcal{V}}^{1}, {\tilde{p }}^{1}\in {\mathcal{P}}^{1}, {\stackrel{\sim }{{\varvec{u}}}}_{s}^{2},{\stackrel{\sim }{{\varvec{v}}}}_{s}^{2},{\stackrel{\sim }{{\varvec{v}}}}_{flt}^{2}\in {\mathcal{V}}_{u}^{2}, {\stackrel{\sim }{{\varvec{v}}}}_{f}^{2} \in {\mathcal{V}}^{2}, {\tilde{p }}^{2} \in {\mathcal{P}}^{2}.$$

48 $${\left({\stackrel{\sim }{{\varvec{v}}}}_{s}^{1}, {\zeta }_{{R}_{s}}^{1}{\rho }_{s}^{*}\frac{\partial {{\varvec{v}}}_{s}^{1}}{\partial t}+ {\zeta }_{{R}_{s}}^{1}{{{\varvec{F}}}_{s}^{1}}^{-T}\nabla {p}^{1}-\left({J}_{s}^{1}- {\zeta }_{{R}_{s}}^{1}\right)\frac{{\mu }_{f}}{{k}_{s}^{1}}{{\varvec{v}}}_{flt}^{1}\right)}_{1} + \mathrm{b}{\left({{\stackrel{\sim }{{\varvec{v}}}}_{s}^{1},\boldsymbol{ }{\varvec{P}}}_{s}^{1}\right)}_{1}=0$$

49 $${\left({\stackrel{\sim }{{\varvec{v}}}}_{s}^{2}, {\zeta }_{{R}_{s}}^{2}{\rho }_{s}^{*}\frac{\partial {{\varvec{v}}}_{s}^{2}}{\partial t}+ {\zeta }_{{R}_{s}}^{2}{{{\varvec{F}}}_{s}^{2}}^{-T}\nabla {p}^{2}-\left({J}_{s}^{2}- {\zeta }_{{R}_{s}}^{2}\right)\frac{{\mu }_{f}}{{k}_{s}^{2}}{{\varvec{v}}}_{flt}^{2}\right)}_{1} + \mathrm{b}{\left({{\stackrel{\sim }{{\varvec{v}}}}_{s}^{2},\boldsymbol{ }{\varvec{P}}}_{s}^{2}\right)}_{2} - {\left({\stackrel{\sim }{{\varvec{v}}}}_{s}^{2} , {{\zeta}}_{{R}_{s}}^{2}\left({{p}^{2}{{{\varvec{F}}}_{s}^{2}}^{-T}+\boldsymbol{ }\frac{1}{{J}_{s}^{2}}{\varvec{P}}}_{mix}^{1}\right){\widehat{{\varvec{n}}}}^{2}\right)}_{\partial {{\widehat{\Omega }}^{2}}_{I}}=0.$$

50 $$\begin{aligned}{\left(\left({J}_{s}^{1}-{\zeta }_{{R}_{s}}^{1}\right){\stackrel{\sim }{{\varvec{v}}}}_{flt}^{1},{\rho }_{f}^{*}\frac{\partial {{\varvec{v}}}_{f}^{1}}{\partial t}+\frac{{J}_{s}^{1}{\rho }_{f}^{*}}{\left({J}_{s}^{1}- {\zeta }_{{R}_{s}}^{1}\right)}{{{\varvec{F}}}_{s}^{1}}^{-1}\left(\nabla {{\varvec{v}}}_{f}^{1}\right){{\varvec{v}}}_{flt}^{1}+{{{\varvec{F}}}_{s}^{1}}^{-T}\nabla {p}^{1}+\frac{{\mu }_{f}}{{k}_{s}^{1}}{{\varvec{v}}}_{flt}^{1}\right)}_{1}\\+b{\left({\stackrel{\sim }{{\varvec{v}}}}_{flt}^{1}, {{\varvec{P}}}_{f}^{1}\right)}_{1}-{\left(\left({{J}_{s}^{1}-{\zeta}}_{{R}_{s}}^{1}\right){\stackrel{\sim }{{\varvec{v}}}}_{flt}^{1}, { p}^{1}{{{\varvec{F}}}_{s}^{1}}^{-T}{\widehat{{\varvec{n}}}}^{1}\right)}_{\partial {{\widehat{\Omega }}^{1}}_{N1}}- {\left(\left({{J}_{s}^{1}-{\zeta}}_{{R}_{s}}^{1}\right){\stackrel{\sim }{{\varvec{v}}}}_{flt}^{1}, \left({ p}^{1}-{p}_{0}\right){{{\varvec{F}}}_{s}^{1}}^{-T}{\widehat{{\varvec{n}}}}^{1}\right)}_{\partial {{\widehat{\Omega }}^{1}}_{N2}}\\- {\left(\left({{J}_{s}^{1}-{\zeta}}_{{R}_{s}}^{1}\right){\stackrel{\sim }{{\varvec{v}}}}_{flt}^{1}, \left({ p}^{1}-{p}_{Robin}\right){{{\varvec{F}}}_{s}^{1}}^{-T}{\widehat{{\varvec{n}}}}^{1}\right)}_{\partial {{\widehat{\Omega }}^{1}}_{N3}}=0.\end{aligned}$$

51 $$\begin{aligned}{\left(\left({J}_{s}^{1}-{\zeta }_{{R}_{s}}^{1}\right){\stackrel{\sim }{{\varvec{v}}}}_{flt}^{1},{\rho }_{f}^{*}\frac{\partial {{\varvec{v}}}_{f}^{1}}{\partial t}+\frac{{J}_{s}^{1}{\rho }_{f}^{*}}{\left({J}_{s}^{1}- {\zeta }_{{R}_{s}}^{1}\right)}{{{\varvec{F}}}_{s}^{1}}^{-1}\left(\nabla {{\varvec{v}}}_{f}^{1}\right){{\varvec{v}}}_{flt}^{1}+{{{\varvec{F}}}_{s}^{1}}^{-T}\nabla {p}^{1}+\frac{{\mu }_{f}}{{k}_{s}^{1}}{{\varvec{v}}}_{flt}^{1}\right)}_{1}\\+b{\left({\stackrel{\sim }{{\varvec{v}}}}_{flt}^{1}, {{\varvec{P}}}_{f}^{1}\right)}_{1} - {\left({\stackrel{\sim }{{\varvec{v}}}}_{flt}^{2} , \left({J}_{s}^{2}-{\zeta }_{{R}_{s}}^{2}\right)\left({{p}^{2}{{{\varvec{F}}}_{s}^{2}}^{-T}\boldsymbol{ }+\boldsymbol{ }\frac{1}{{J}_{s}^{2}}{\varvec{P}}}_{mix}^{1}\right){\widehat{{\varvec{n}}}}^{2}\right)}_{\partial {{\widehat{\Omega }}^{2}}_{I}}=0.\end{aligned}$$

52 $${\left({\stackrel{\sim }{{\varvec{v}}}}_{f}^{a}, {J}_{s}^{a}{{\varvec{v}}}_{flt}^{a}-\left({J}_{s}^{a}- {\zeta }_{{R}_{s}}^{a}\right)\left({{\varvec{v}}}_{f}^{a}- {{\varvec{v}}}_{s}^{a}\right)\right)}_{a}=0, \quad for\; a=1, 2.$$

53 $$b{\left({{\varvec{v}}}_{s}^{a}+ {{\varvec{v}}}_{flt}^{a}, {\tilde{p }}^{a}{{{\varvec{F}}}_{s}^{a}}^{-T}\right)}_{a} =0, \quad for\; a=1, 2.$$

54 $${\left({\stackrel{\sim }{{\varvec{u}}}}_{s}^{a}, \frac{\partial {{\varvec{u}}}_{s}^{a}}{\partial t}- {{\varvec{v}}}_{s}^{a}\right)}_{a}=0, \quad for\; a=1, 2.$$

In Eq. (50), $\partial {{\widehat{\Omega }}^{1}}_{N1}$, $\partial {{\widehat{\Omega }}^{1}}_{N2}$ and $\partial {{\widehat{\Omega }}^{1}}_{N3}$ are the boundaries to ${\widehat{\Omega }}^{1}$ at $y= -100$, $y= 100$ and $z= 0$, respectively.

These weak form equations were converted to their component form using Wolfram Mathematica. The equations were implemented using the weak form PDE module in COMSOL Multiphysics. A mixed-finite element model was used with second order Lagrange polynomials for all variables except pressure, which used a first order Lagrange polynomial. The initial conditions were set to zero value for all variables. A baseline time-dependent problem was solved, where the magnitude of the traction on $\partial {{\widehat{\Omega }}^{1}}_{N2}$ was ramped from 0 to ${p}_{0}$ in 0.1 s, with no arteriolar dilation. The baseline problem was run for 0.5 s, to reach a steady state (the changes in filtration velocity less than 0.01%). The simulations with vasodilation were then performed with the initial conditions set to the last timestep of the baseline model, and the outputs were saved for 201 time points between 0.5 and 10.5. Second order backward difference formula (BDF), with a timestep of 0.0025 s was used to solve the time-dependent problems.

### Fluid particle tracking

For tracking the motion of fluid, we need the fluid particle velocities in the computational frame. Therefore, we used the fluid velocities and the displacement fields calculated using the finite element model and calculated the fluid particle velocity in the computational frame. The equation for fluid particle velocity in the computational frame (Eq. 55) was derived in our previous publication [62] and is valid for both domains. The fluid velocities for both the domains were exported into a text format using COMSOL Multiphysics for all the points on the computational grid for the 201 time points to be used for fluid particle tracking.

55 $${\dot{X}}_{f}= {{{\varvec{F}}}_{s}^{a}}^{-1}\left({{\varvec{v}}}_{f}^{a}- {{\varvec{v}}}_{s}^{a}\right) \;\text{for}\;a=1, 2.$$

A grid of equally spaced points (Fig. 3a) was created using Altair Hypermesh. Similarly, grids and meshed were created for the boundaries of the two domains and the surface representing the arteriolar wall. The grids and meshes were converted to text format using Microsoft Excel.

The fluid particle velocities, along with the grids and meshes were imported into MATLAB. The data was repeated along the time axis to calculate the fluid particle trajectories for 60 s. The particle trajectories were calculated by interpolating the imported velocity and using a backward Euler integration scheme.

### Non-dimensional numbers

The field equations and the data set of the problem studied in this paper produce a framework with a very rich structure. A full dimensional analysis guided by the $\Pi$-theorem (cf. [6]), would require an extensive study of its own. Here we discuss the nondimensional groups that we believe are most pertinent to the presentation of our results. Specifically, we do not discuss any wave propagation phenomena linked to the elastic response of the solid phase. We point out that Eqs. (12), (13), and (15) have the expression ${J}_{s}^{a}- {\upzeta }_{{R}_{s}}^{a}$ in several of their terms. If ${J}_{s}^{a}- {\upzeta }_{{R}_{s}}^{a}$ were equal to zero at some location in the solution’s domain, we would have the disappearance of the fluid phase at said location and the field equations would change their type. The data used in our simulations never caused such an occurrence (changes in ${J}_{s}^{a}$ are on the order of 1%) and therefore we do not consider it in this discussion. Instead, we focus on some elements of Eq. (13) and the flow regimes that we do have in our simulations, with estimates that are generally applicable across all of the cases we studied. Equation (13) (with the definition of viscous stress in Eq. (15)) has features typical of the Navier–Stokes and the Darcy flow equations. Of the various nondimensional groups that can arise from the dimensional analysis of Eq. (13), here we focus on the Darcy, Womersley, and Reynolds numbers. The Darcy number ${\text{Da}}={k}_{s}/{L}^{2}$, where ${k}_{s}$ is the Darcy permeability and $L$ is a relevant length scale. Physically, we view the Darcy number describing the relative importance of the terms $\frac{{\mu }_{f}}{{k}_{s}^{a}}{{\varvec{v}}}_{flt}^{a}$ and $\nabla\!\cdot\!{{\varvec{P}}}_{f}^{a}$ in Eq. (13). The values of Darcy permeability we used ranged from $2\times {10}^{-15} {\text{m}}^{2}$ to $2\times {10}^{-12} {\text{m}}^{2}$ depending on the position of points along the $z$-axis. Among the relevant length scales, we choose the smallest, namely the amplitude of the wall motion (from $1.5\,\upmu\mathrm{m}$ up to 40% of arterial radius, $<5\,\upmu\mathrm{m}$). The associated range of the Darcy number is therefore of the $0.88\times {10}^{-3}$ to about 0.8, which we feel justifies (perhaps with an excess of caution) the use of a Darcy-Brinkman model rather than a mere Darcy flow model (i.e., without the term originating from ${{\varvec{\sigma}}}_{f}^{a}$), is able to adequately represent flow across said range of values. As far as the Womersley number [36] is concerned it is an indicator of the relative magnitude of inertia and viscous forces. Again, using Eq. (13) as the backdrop, we define the Womersley number the following manner: ${\text{Wo}}={\rho }_{f}\left(\left|\left|\varvec{v}_{f}\right|\right|/\tau \right)/\left[\left({\mu }_{f}/{k}_{s}\right)\left|\left|\varvec{v}_{flt}\right|\right|\right]={\rho }_{f}^{*}{k}_{s}{\tau }^{-1}{\mu }_{f}^{-1}\left|\left|\varvec{v}_{f}\right|\right|/\left|\left|\varvec{v}_{f}-\varvec{v}_{s}\right|\right|$, where $\tau$ is an appropriate the time scale, and where we have used the definitions in Eqs. (3) and (6). By and large, across all our simulations and almost independently of time and position the ratio $\left|\left|\varvec{v}_{f}\right|\right|/\left|\left|\varvec{v}_{f}-\varvec{v}_{s}\right|\right|$ is less than 2, in fact close to 1. For simplicity, and with the goal of producing a conservative upper bound for Wo, we will therefore choose 10 as a very generous (perhaps excessive) upper bound for ratio $\left|\left|\varvec{v}_{f}\right|\right|/\left|\left|\varvec{v}_{f}-\varvec{v}_{s}\right|\right|$ and we will choose the largest value of ${k}_{s}$ introduced earlier, namely $2\times {10}^{-12} {\text{m}}^{2}$. As far as $\tau$ is concerned, and again with the purpose of generating a conservative upper bound for Wo, we choose $1\,\mathrm{s}$ (cf., e.g., Fig. 3), which is the time interval over which the maximum wall motion occurs. In summary, we have we find that Wo is less than $2\times {10}^{-5}$. This estimate implies that effects due to inertia are strongly dominated by viscous effects and therefore could have possibly been neglected in our equations. The prominence of viscous forces is also apparent in our estimates of relevant Reynolds numbers. We defined Reynolds numbers based on the axial flow through the PVS at the surface of the brain ($z=150\,\upmu\mathrm{m}$), the mid-plane of the model ($z=75\,\upmu\mathrm{m}$) and at the bottom of the PVS ($z=0$). The spatial-average flow velocity through the cross-section was calculated using the flowrate and the fluid area (Eqs. 56, 57). A characteristic length of highest PVS thickness in the model (${t}_{PVS}= 8\,\upmu\mathrm{m}$) was used to calculate the Reynolds numbers.

56 $${Q}_{z= 150, 75, 0}= \underset{z=150, 75, 0}{\overset{}{\int }}{J}_{s}^{1}{{\varvec{v}}}_{flt}^{1}\cdot {{{\varvec{F}}}_{s}^{1}}^{-T}\widehat{z}, {A}_{z= 150, 75, 0}= \underset{z=150, 75, 0}{\overset{}{\int }}{J}_{s}^{1}{\zeta }_{{R}_{s}}^{1}.$$

57 $${v}_{Avg, z}= \frac{{Q}_{z}}{{A}_{Z}}.$$

58 $${Re}_{z}= \frac{{\rho }_{f}{v}_{Avg, z}{t}_{PVS}}{{\mu }_{f}}.$$

As shown in Additional file 1: Fig. S8, the values of the Reynolds numbers observed in our calculations confirm that the flow is dominated by viscous forces.

As mentioned earlier in the paper, we chose Peclet numbers to present some of our results even though Peclet numbers do not arise from a formal dimensional analysis of our field equations. As such, we do not view Peclet numbers as flow indices. Rather, we view them as a way, admittedly unconventional, to bring into the discussion of our result considerations on transport driven by a process of Fickian diffusion as opposed to a process of Darcy-like diffusion, promoted by the pressure gradients engendered by arterial wall motion.

Two Peclet numbers were defined to compare the convective and diffusive transport driven by vasodilation. We used non-traditional definitions for the Peclet numbers to represent the flow of PVS fluid through the ECS. A characteristic length of ${L}_{A-V}=150\,\upmu\mathrm{m}$ [2, 51] was used to represent the distance between arteriolar and venous PVS. The diffusion coefficient of amyloid-β ${D}_{a\beta }$ was used to calculate the Peclet numbers. Effectively, the Peclet numbers defined here represent the ratio of time taken for diffusive and convective transport of amyloid-β from arterial to venous PVS.

We considered two possible pathways of fluid transport from arteriolar to venous PVS. The axial Peclet number (${Pe}_{a}$), was defined based on the flow through the bottom of the PVS ($z=0$), and represents pumping by arteriolar wall movements similar to peristaltic pumping..

59 $${Pe}_{a}= \frac{{v}_{Avg, z=0}{L}_{A-V}}{{D}_{a\beta }}.$$

The radial Peclet number was defined to represent fluid penetration into the brain parenchyma and was based on the relative velocity of the fluid with respect to the solid in the radial direction into the ECS, in the immediate vicinity of the PVS-ECS interface (Eq. 60). In Eq. (60), $\widehat{{\varvec{r}}}$ is the unit normal vector in the radially outward direction and $\lambda$ is the tortuosity of the ECS.

60 $${Pe}_{r}= \frac{{{\varvec{v}}}_{flt}^{2}\cdot \widehat{{\varvec{r}}}\boldsymbol{ }{L}_{A-V}}{{D}_{a\beta }/{\lambda }^{2}}.$$

## Supplementary Information

**Additional file 1**: **Fig. S1**. 2D poroelastic model demonstrates the difference between SAS and ECS fluid exchange during arteriolar dilation. **Fig. S2**. Filtration velocity for temporally symmetric and asymmetric dilation. **Fig. S3**. Dilation of the brain tissue in the model at the PVS-ECS interface in the radial direction **b-c** and in the vertical direction **e-f.** The three locations where the displacement was calculated is shown in **a. **The blue line in all the subplots is the arteriolar wall dilation in the radial direction. **Fig. S4**. Directional fluid flow from the PVS into the ECS driven by vasodilation is not an artifact of the imposed pressure difference across the SAS. **Fig. S5**. PVS fluid penetration into the ECS increases with increased brain fluid permeability (${k}_{s}^{2}$). **Fig. S6**. PVS fluid penetration into the ECS is higher for low frequency vasodilation. **Fig S7**. The area under the dilation curve, not the maximum dilation amplitude, is an indicator of directional PVS fluid flows into the ECS. **Fig S8**. The spatial-average axial fluid velocity and Reynolds number at different depths in the model for **(b)** 20% asymmetric dilation with default parameters,** (c)** 20% asymmetric dilation with a small (0.001 mmHg) pressure difference across the SAS, **(d)** 20% symmetric dilation with default parameters and **(e)** 40% dilation with simulated sleep state (increased ECS permeability and porosity). Negative values indicate flow in the negative-z direction and into the PVS, in the direction of blood flow. **(a)** shows the cross sections where the average velocity was calculated.

## Data Availability

Simulation files are available. The step files are available on GitHub: https://github.com/kraviteja89/poroelastic3DPVS.
