# Supplementary material for: Arterial vasodilation drives convective fluid flow in the brain: a poroelastic model
Source: Fluids Barriers CNS. 2022 May 15;19:34. doi: 10.1186/s12987-022-00326-y (PMC9107702; doi:10.1186/s12987-022-00326-y)
Supplement: Supplementary file 1 — Additional file 1: Fig. S1. 2D poroelastic model demonstrates the difference between SAS and ECS fluid exchange during arteriolar dilation. Fig. S2. Filtration velocity for temporally symmetric and asymmetric dilation. Fig. S3. Dilation of the brain tissue in the model at the PVS-ECS interface in the radial direction b-c and in the vertical direction e-f. The three locations where the displacement was calculated is shown in a. The blue line in all the subplots is the arteriolar wall dilation in the radial direction. Fig. S4. Directional fluid flow from the PVS into the ECS driven by vasodilation is not an artifact of the imposed pressure difference across the SAS. Fig. S5. PVS fluid penetration into the ECS increases with increased brain fluid permeability (${k}_{s}^{2}$ks2). Fig. S6. PVS fluid penetration into the ECS is higher for low frequency vasodilation. Fig S7. The area under the dilation curve, not the maximum dilation amplitude, is an indicator of directional PVS fluid flows into the ECS. Fig S8. The spatial-average axial fluid velocity and Reynolds number at different depths in the model for (b) 20% asymmetric dilation with default parameters, (c) 20% asymmetric dilation with a small (0.001 mmHg) pressure difference across the SAS, (d) 20% symmetric dilation with default parameters and (e) 40% dilation with simulated sleep state (increased ECS permeability and porosity). Negative values indicate flow in the negative-z direction and into the PVS, in the direction of blood flow. (a) shows the cross sections where the average velocity was calculated. [file 12987_2022_326_MOESM1_ESM.docx]

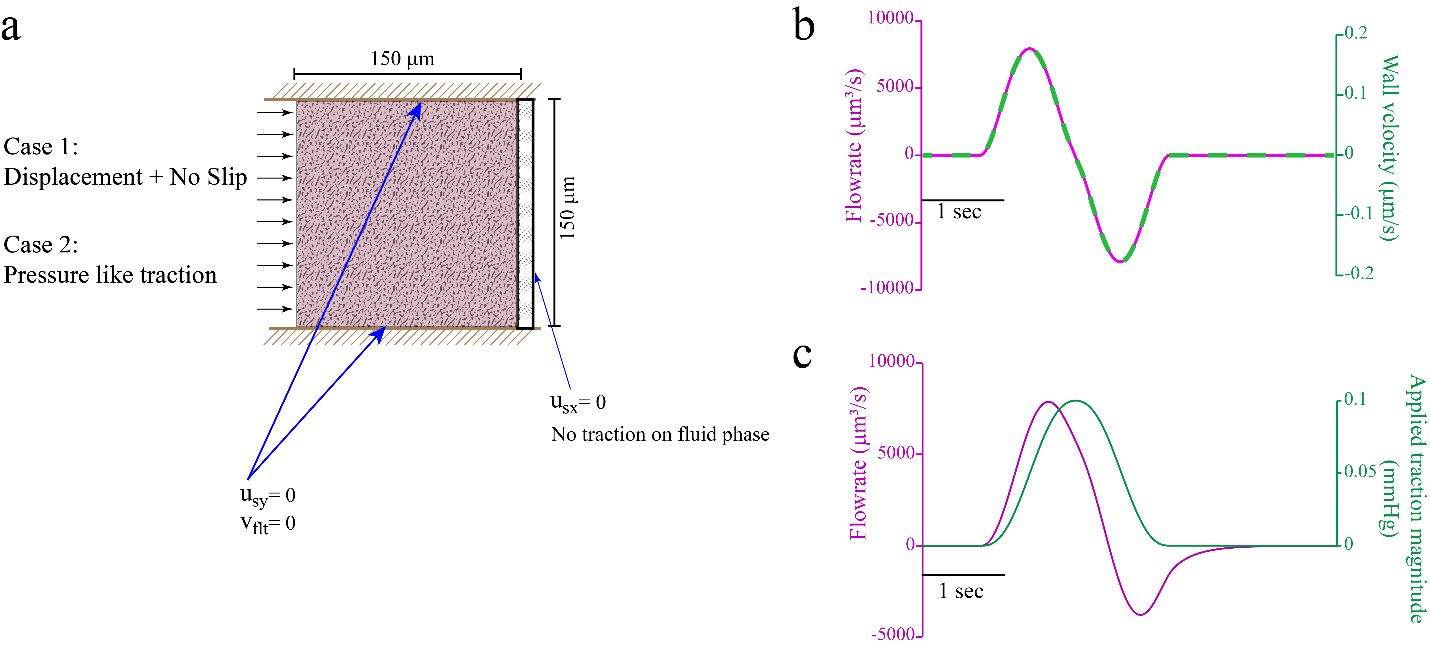


Fig. S1: 2D poroelastic model demonstrates the difference between SAS and ECS fluid exchange during arteriolar dilation.

**a.** 2D Poroelastic model dimensions and boundary conditions. All other parameters were the same as the ones used for the brain tissue in the rest of the article. **b.** Flow rate through the right edge has the same waveform as the wall velocity of the left edge for Case 1, where a displacement boundary condition was imposed on the left edge along with a no-slip condition. **c.** Flow rate through the right edge lags the changes in the pressure-like traction at the left edge for Case 2, where a pressure-like traction was applied on the left edge.

Case 1, where a direct displacement boundary condition was imposed represents flow through the SAS during arteriolar dilation. Case 2 represents flow through the ECS, where fluid flow is induced by pressure changes in the PVS.

Note: The flow rate was calculated by assuming a $150\mu m$ thickness perpendicular to the plane.


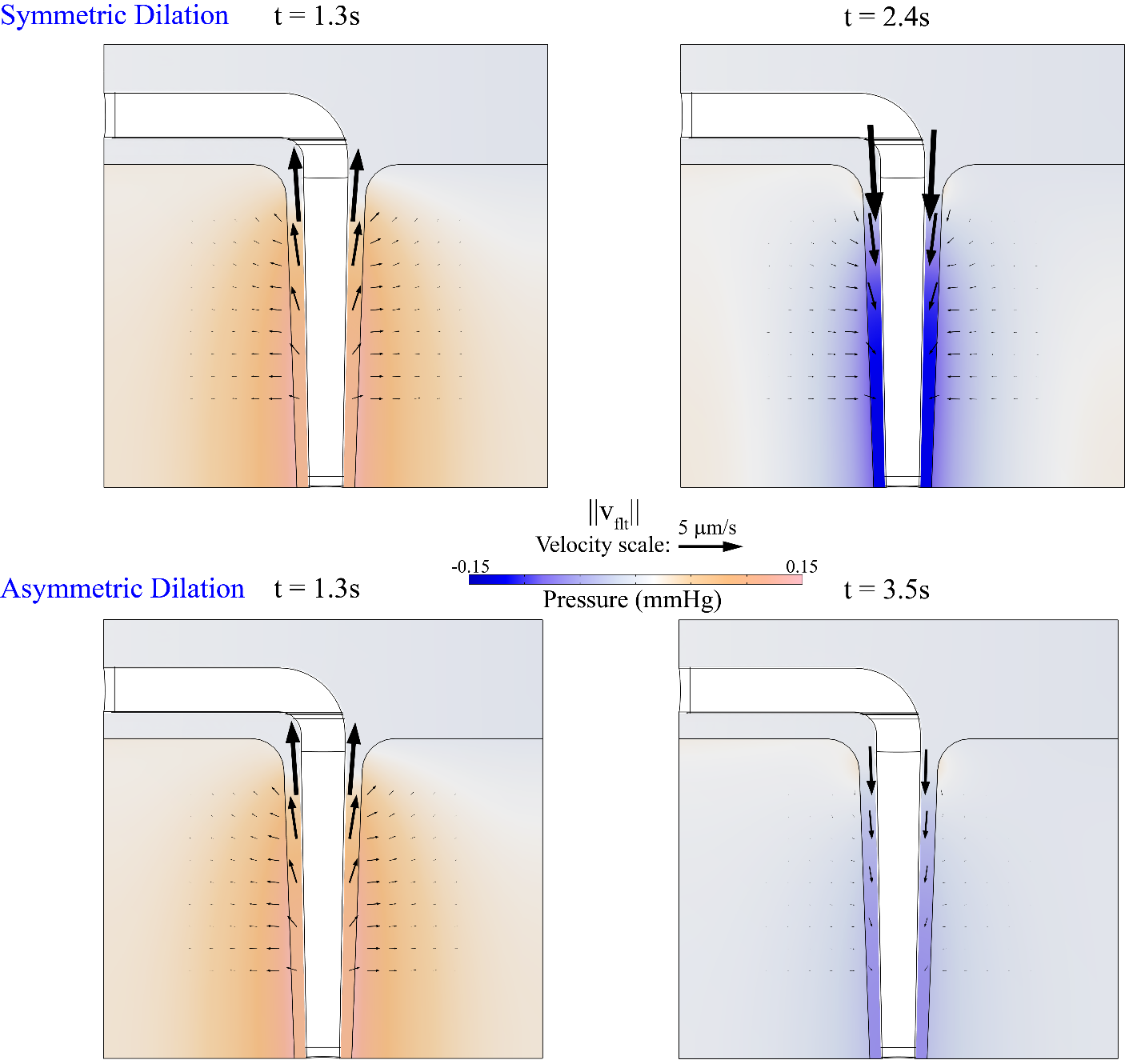


Fig. S2: Filtration velocity for temporally symmetric and asymmetric dilation.

The pressure and filtration velocity in the PVS and the ECS at the times of maximum radially outward and inward arteriolar wall velocity for symmetric (top) and asymmetric (bottom) dilation. The colors show the pressure value in mmHg and the arrows show the magnitude and direction of the filtration velocity. Fewer rows of arrows were used in the PVS to avoid overlapping arrows. The continuity of filtration velocity across the two regions is best demonstrated by the bottom most row, where the fluid flows more in the radial direction than the axial direction.


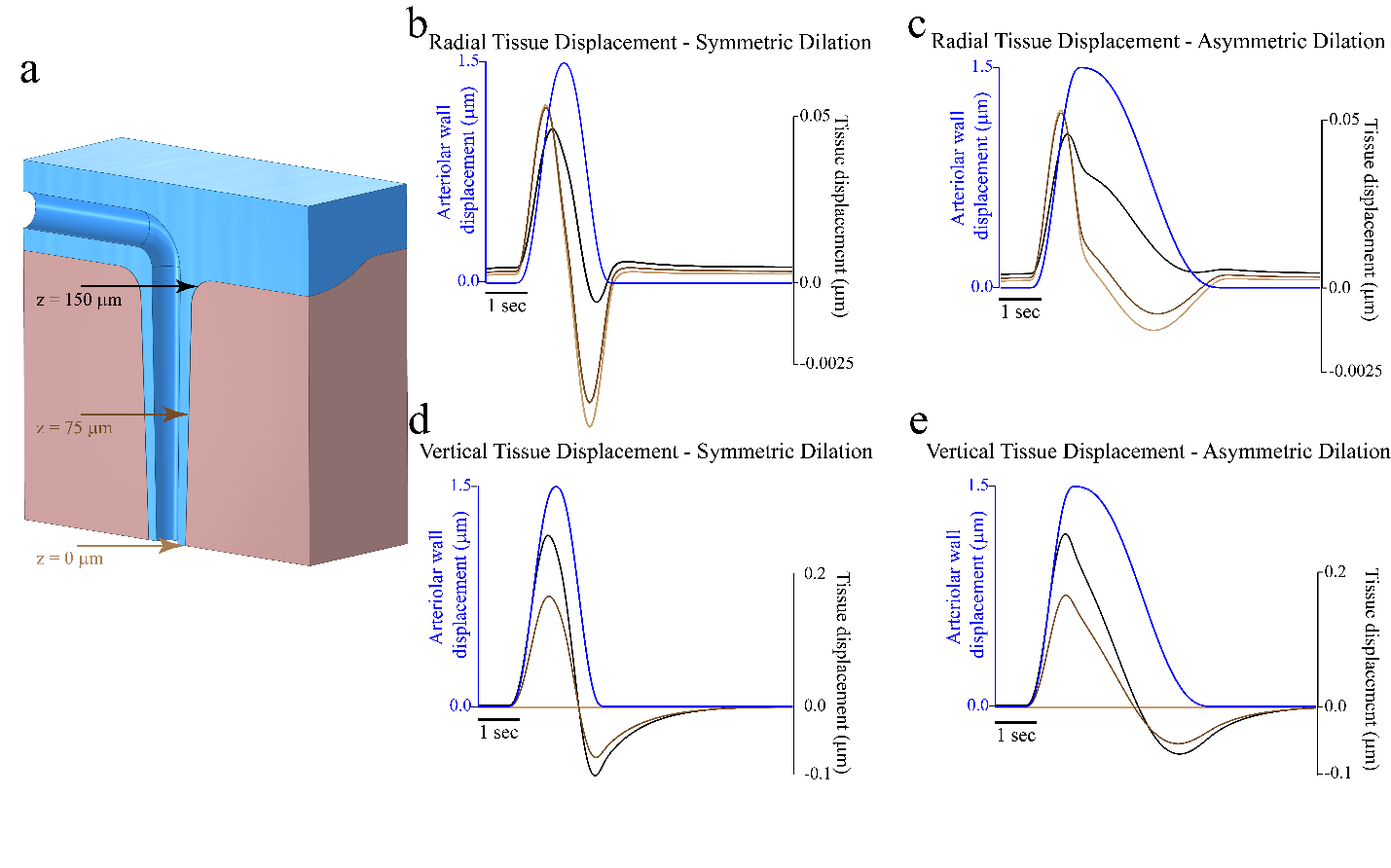


Fig. S3: Dilation of the brain tissue in the model at the PVS-ECS interface in the radial direction **b-c** and in the vertical direction **e-f.** The three locations where the displacement was calculated is shown in **a.** The blue line in all the subplots is the arteriolar wall dilation in the radial direction.


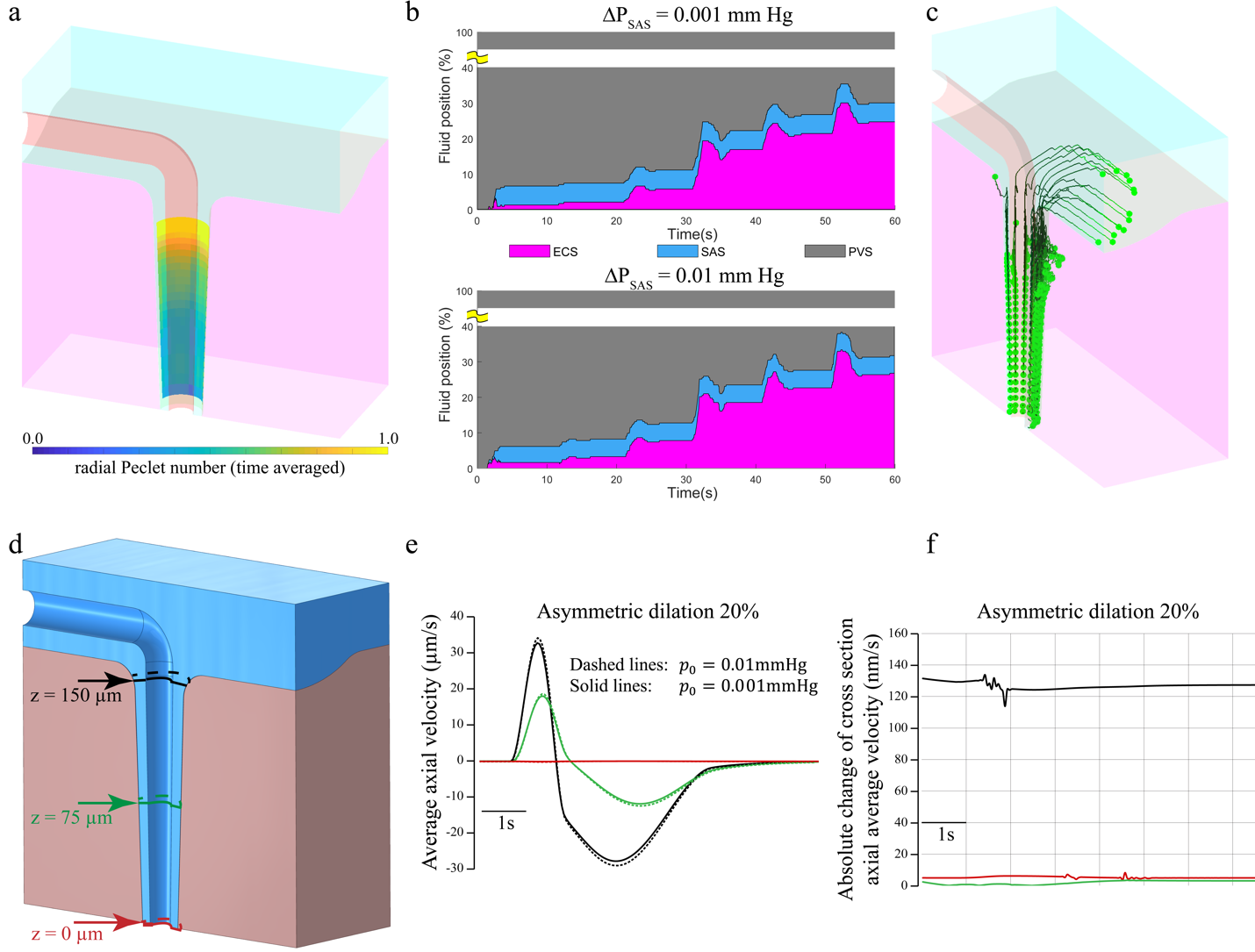


Fig. S4: Directional fluid flow from the PVS into the ECS driven by vasodilation is not an artifact of the imposed pressure difference across the SAS

The simulations with the asymmetric vasodilation (bottom part of Fig. 3) were repeated with a smaller pressure difference $p_{0}=0.001 \text{mmHg}$ across the SAS (**a**–**c**). In (a) we report the plot of the radial Peclet number at the PVS–ECS interface, with the radial Peclet number averaged over 10s of simulation with a single 5-second-long vasodilation event. The plot in (**a**) points to the fact that directional fluid flow driven by the temporally asymmetric waveform of functional hyperemia is not an artifact of the fluid flow in the SAS. This is confirmed by examining the PVS fluid position **(b)** and trajectories **(c)** in a particle tracking simulation performed for 60s, where a single vasodilation event is repeated once every 10s. To reinforce our claim more strongly, and to facilitate a comparison with the results in Fig. 3, (**e**) reports the averaged axial velocity for both the cases when $p_{0}=0.001 \text{mmHg}$ (solid lines) and $p_{0}=0.01 \text{mmHg}$ (dashed lines) computed at the three cross-sections indicated in (**d**). The two cases overlap substantially at this resolution. For this reason, the magnitude of the difference of the averaged axial velocity in the two pressure conditions has been plotted in (**f**), which shows that this difference is on the order of nm/s, thus further substantiating the claim that the directional flow in the PVS into the ECS driven by vasodilation is not an artifact of the pressure difference across the SAS. We note that the results in (**e**) are reported Fig. S8 for the two cases separately.


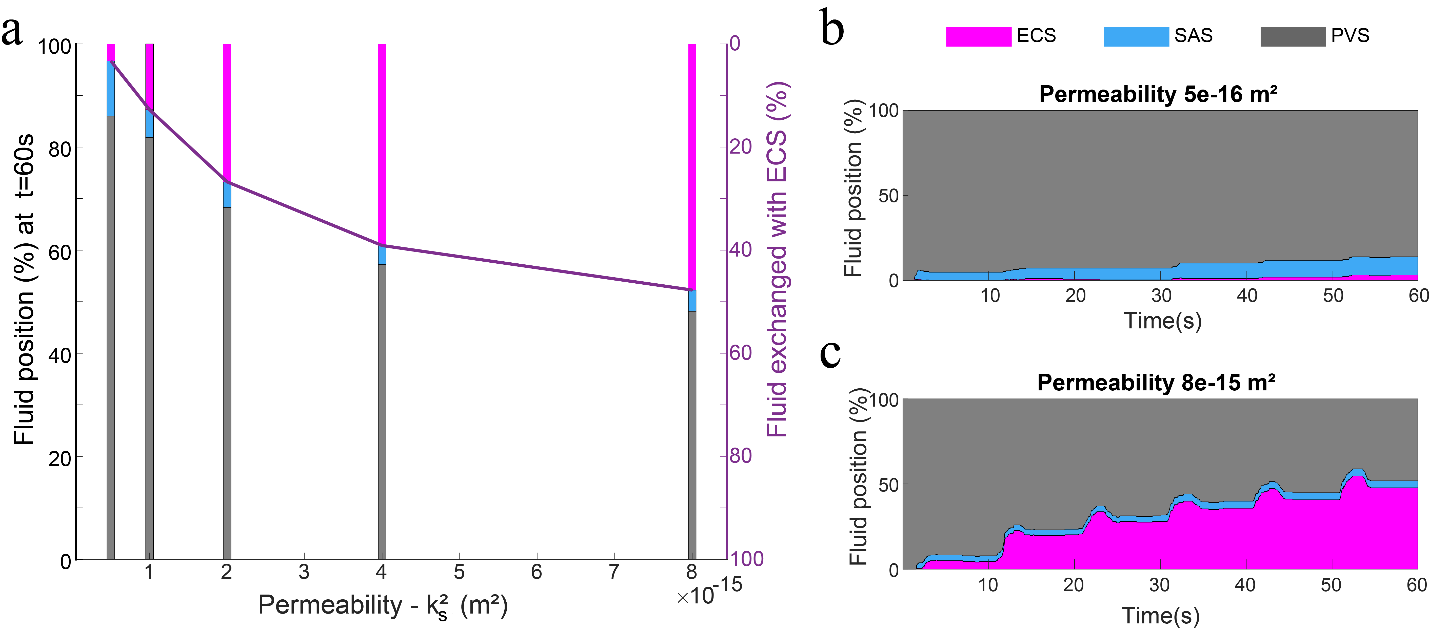


Fig. S5: PVS fluid penetration into the ECS increases with increased brain fluid permeability ($k_{s}^{2}$).

**a.** PVS Fluid distribution at the end of a 60 second fluid particle tracking simulation. The fluid exchanged with ECS (magenta) increases with increased permeability, while fluid exchanged with SAS (blue) decreases. **b.** and **c.** Show the fluid distribution during the 60 seconds for $k_{s}^{2}=0.5 \times{10}^{-15}m^{2}$ and $k_{s}^{2}=8.0 \times{10}^{-15}m^{2}$ respectively.


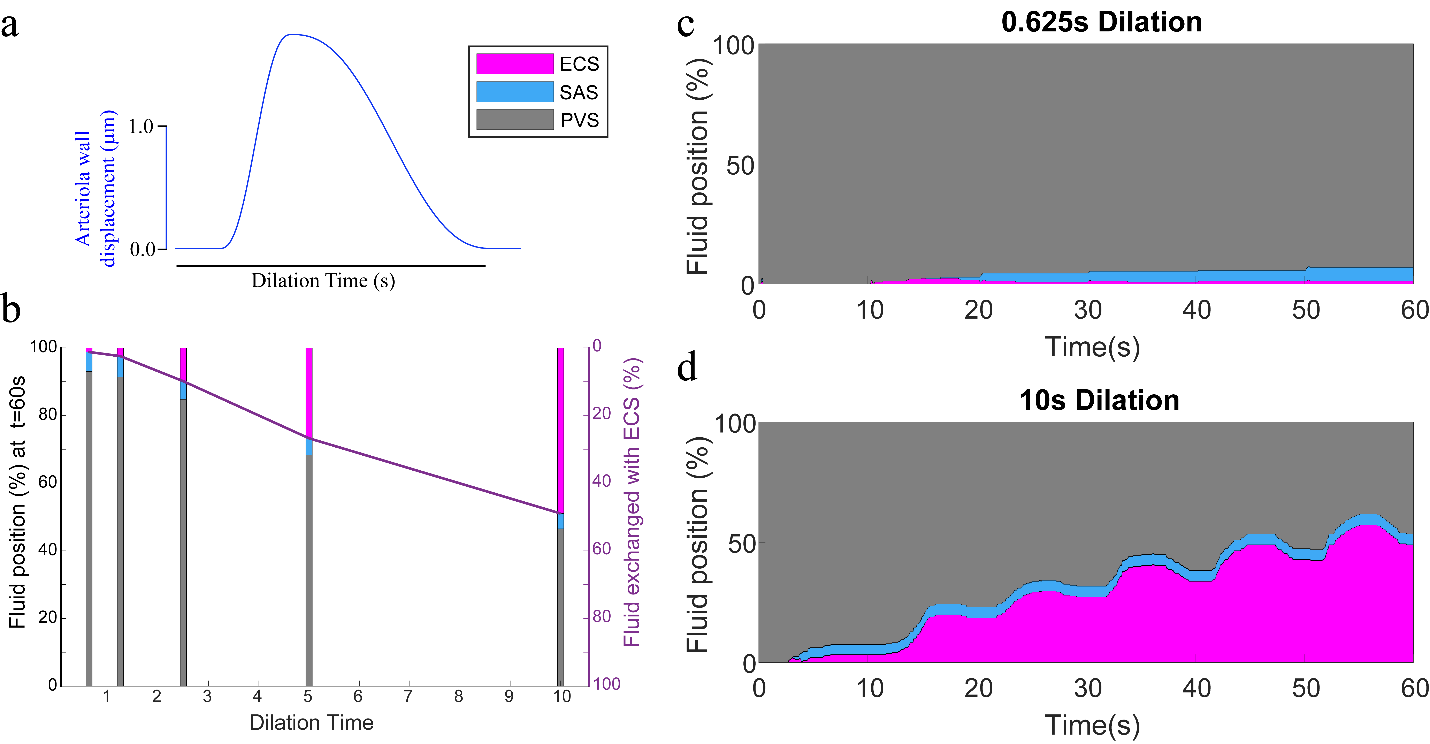


Fig. S6: PVS fluid penetration into the ECS is higher for low frequency vasodilation.

a. The arteriolar dilation waveform showing the dilation time. One dilation event was used for 10 seconds of simulation. b. PVS Fluid distribution at the end of a 60 second fluid particle tracking simulation. The fluid exchanged with ECS (magenta) is higher for slower dilation, while fluid exchanged with SAS (blue) does not change appreciably with dilation frequency. **c.** and **d.** Show the fluid distribution during the 60 seconds for dilation time of 0.625s and 10s respectively.


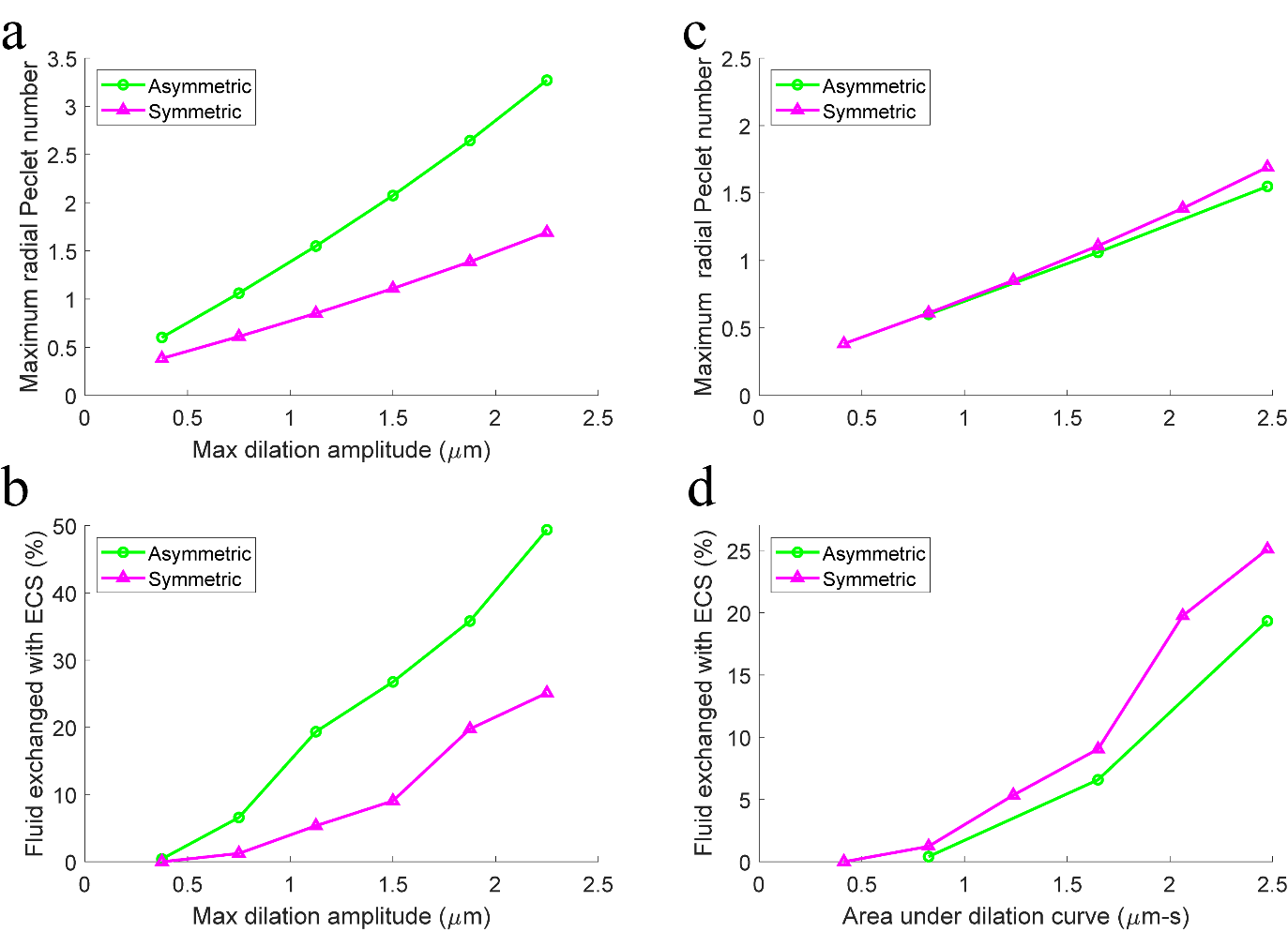


Fig S7: The area under the dilation curve, not the maximum dilation amplitude, is an indicator of directional PVS fluid flows into the ECS.

**a.** and **b.** show that the maximum time-averaged radial Peclet number (${Pe}_{r}$) and PVS fluid exchanged with the ECS (over 60 seconds with one dilation per 10 seconds), which are measures of directional PVS fluid flow into the ECS, are appreciably affected by the on the waveform of the vasodilation for the same peak dilation value.

**c.** and **d.** show that the directional PVS fluid flow into the ECS is not appreciably affected by the waveform, when the area under the dilation curve is kept the same.


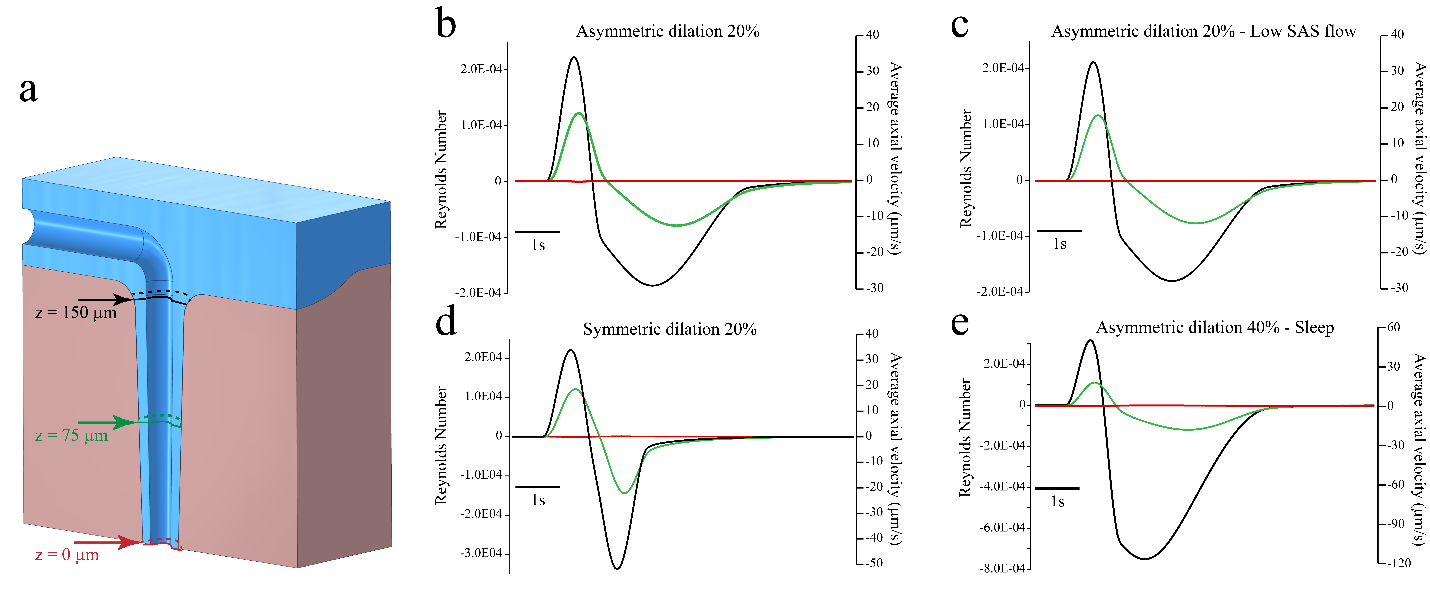


Fig S8: The spatial-average axial fluid velocity and Reynolds number at different depths in the model for **(b)** 20% asymmetric dilation with default parameters, **(c)** 20% asymmetric dilation with a small (0.001 mmHg) pressure difference across the SAS, **(d)** 20% symmetric dilation with default parameters and **(e)** 40% dilation with simulated sleep state (increased ECS permeability and porosity). Negative values indicate flow in the negative-z direction and into the PVS, in the direction of blood flow. **(a)** shows the cross sections where the average velocity was calculated.
